# Metamorphic aerial robot capable of mid-air shape morphing for rapid perching

**DOI:** 10.1038/s41598-022-26066-5

**Published:** 2023-01-23

**Authors:** Peter Zheng, Feng Xiao, Pham Huy Nguyen, Andre Farinha, Mirko Kovac

**Affiliations:** 1 Aerial Robotics Laboratory, Department of Aeronautics, Imperial College London, London, SW7 2AZ UK; 2 The Grantham Institute-Climate Change and the Environment, Imperial College London, London, SW7 2AZ UK; 3 Laboratory of Sustainability Robotics, Swiss Federal Laboratories of Materials Science and Technology, 8600 Dübendorf, Switzerland

**Keywords:** Mechanical engineering, Forestry

## Abstract

Aerial robots can perch onto structures at heights to reduce energy use or to remain firmly in place when interacting with their surroundings. Like how birds have wings to fly and legs to perch, these bio-inspired aerial robots use independent perching modules. However, modular design not only increases the weight of the robot but also its size, reducing the areas that the robot can access. To mitigate these problems, we take inspiration from gliding and tree-dwelling mammals such as sugar gliders and sloths. We noted how gliding mammals morph their whole limb to transit between flight and perch, and how sloths optimized their physiology to encourage energy-efficient perching. These insights are applied to design a quadrotor robot that transitions between morphologies to fly and perch with a single-direction tendon drive. The robot’s bi-stable arm is rigid in flight but will conform to its target in 0.97 s when perching, holding its grasp with minimal energy use. We achieved a $30\%$ overall mass reduction by integrating this capability into a single body. The robot perches by a controlled descent or a free-falling drop to avoid turbulent aerodynamic effects. Our proposed design solution can fulfill the need for small perching robots in cluttered environments.

## Introduction

**Figure 1:**
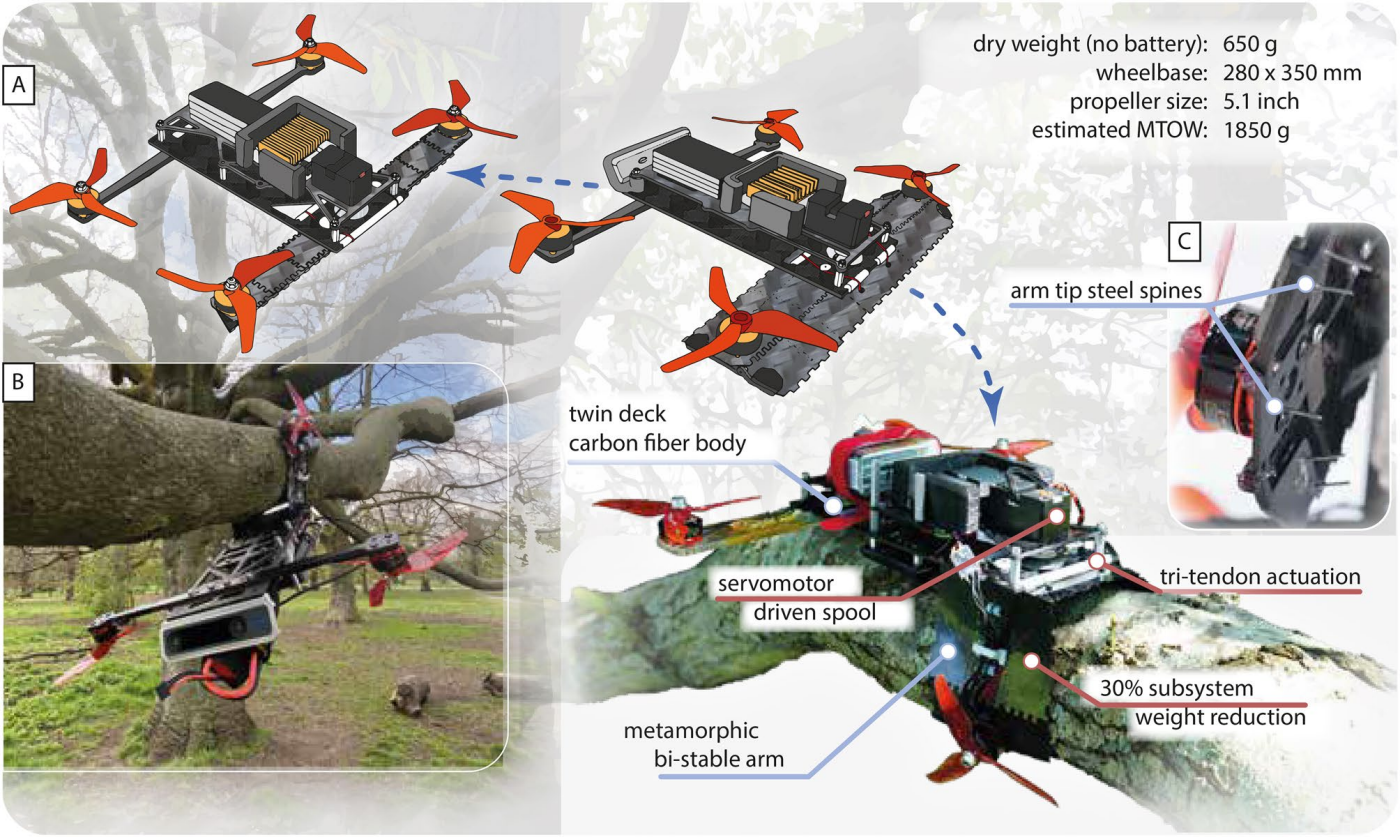
The metamorphic perching quadrotor robot. (**A**) Perching mission cycle with the drawings of the robot in the locked, unlocked, and perched state. (**B**) A photograph of the prototype robot perching upside down on an English oak tree. (**C**) The embedded steel spines across the flat unlocked arm.

The parts of the forest hidden from view are often the most important areas for the conservation management of endangered species and plants^1,2^. While satellites and aircraft can image the forest from above, the leafy treetops can form a barrier. The canopy can obstruct the view to the forest floor and block access to soundscapes from above the treetop. In denser forests, there are also microclimate enclaves that can vastly differ from the climate above the canopy^3^. Multirotor unmanned aerial vehicles (UAV) have been shown to be well-suited for imaging above forest canopies and areas with low vegetation height^4,5^. However, there are limited solutions for remote sensor deployment in these confined spaces^6,7^, especially within a forest^8^.

Forest-dwelling aerial robots need to have high endurance, be small to penetrate deep into a dense forest, and be adaptable to constantly changing environments. Of these requirements, high flight endurance and small robot size are direct trade-offs due to the aerodynamics of propellers. But, for environmental sensing, data can be gathered when the robot is stationary. An environmental sensing mission can be separated into phases of flights within the forest and rest periods for the robot to take sensor readings. Therefore, we propose a metamorphic perching aerial robot that is small and capable of using its multirotor arms to grasp onto tree branches (Fig. 1).

Perching between intermittent movement from tree to tree has often been highlighted as a method to save energy, escape predators, and take cover from errant weather for aerial robots and their biological counterparts^9^. Perching and resting also allow these creatures to be placed at an advantageous position for surveillance^9,10^. Animals, such as sloths and koalas, have also shown their ability to retain, recover, and maintain homeostasis while resting on trees^11^. Furthermore, the sloth’s arm muscle structure is optimized to retract and perch firmly^12^, saving weight and energy consumption by having minimal abducting muscles.

Bioinspired principles have allowed roboticists to reimagine new strategies to approach adaptable perching robot designs^13,14^. There are mechanically activated gecko-inspired, fiber-based dry adhesives that utilize van der Waals forces^15,16^. Alternatively, there are mechanical interaction-based solutions that rely on surface friction and interlocking. Preloaded deployable spikes and passive microspines can perch on rough surfaces^17–20^. Spider-web-inspired methods, utilizing string entanglement or magnetic anchors on ferromagnetic surfaces, are used to perch and suspend the robot in mid-air^21,22^. Specifically targeting cylindrical perch sites, such as tree branches and pipes, active and passive avian-inspired graspers are deployed^20,23–30^. They envelop the target with a claw-like grip and thus are classed as mechanical methods.

Currently, the aforementioned perching methods add additional modules and would increase a drone’s weight^31,32^. Therefore, a compromise between the mission endurance, which includes the time when the robot is perched, and the flight time must be struck. An ideal aerial robotic solution would be a platform that can intelligently transform and adapt its body to perch on various sized and shaped structures without any penalties.

Nature has given robotics various strategies to integrate these mechanisms within a monolithic body, as well as providing bioinspired mechanisms mentioned above. One form of such strategy is metamorphosis. Metamorphosis allows the organism to innately take on various forms, doing so to adapt to its targeted task and/or environment^33^. This transformation of an organism’s body during its life cycle allows the specie to adapt to various ecological niches^34,35^. This improves the survival rate of species which now can depend on different food resources, habitats, and competition^36^. Dragonflies, for example, metamorphose from a larval stage to acquire the ability to fly as an adult^37^. Amphibians like frogs and some salamanders also transform from being solely aquatic to being able to operate in both aquatic and terrestrial habitats^33^.

More recently, the concept of metamorphosis and metamorphic mechanisms have played a key role in the development of multi-modal robotic designs^38^. These metamorphic robots can adapt and perform different tasks and operate in varying environments, such as water, air, and land^39–44^. Aerial robots can use metamorphosis to transition from air to water^45,46^, or from ground to air^47,48^. Metamorphosis has also diversified and augmented the functionality of aerial-only robotic platforms. Aerial robotic platforms can modify their structure during flight to go through different sized apertures^49,50^, protect themselves from collisions^51,52^, or grasp objects^53,54^. This idea can be extended further by using adaptable and programmable structures that can be formed from origami folding or soft materials^55–66^.

Applying metamorphic design to perching mechanisms on aerial robots elegantly fulfills the need for compact long endurance remote sensing platforms. Our proposed robot morphs its multirotor arms between different shape primitives to perform adaptable whole-body grasping over various sized and shaped surfaces while being resistant against wind disturbances. The robot offers a shared functionality between flight and perching, negating the weight and size penalty of any additional grasping module.

In this paper, we present a robotic arm capable of morphing between rigid and compliant modes. Self-locking, telescoping beam structures have been shown to withstand axial and bending loads^67^. However, by employing origami-based self-locking on a triangular cross-section beam design, we realized a side-folding metamorphic arm which is able to resist positive and negative bending loads. And unlike soft morphing or single-morphology articulated arms^68^, our metamorphic arm is sufficiently rigid across the full multirotor flight envelope.

**Figure 2:**
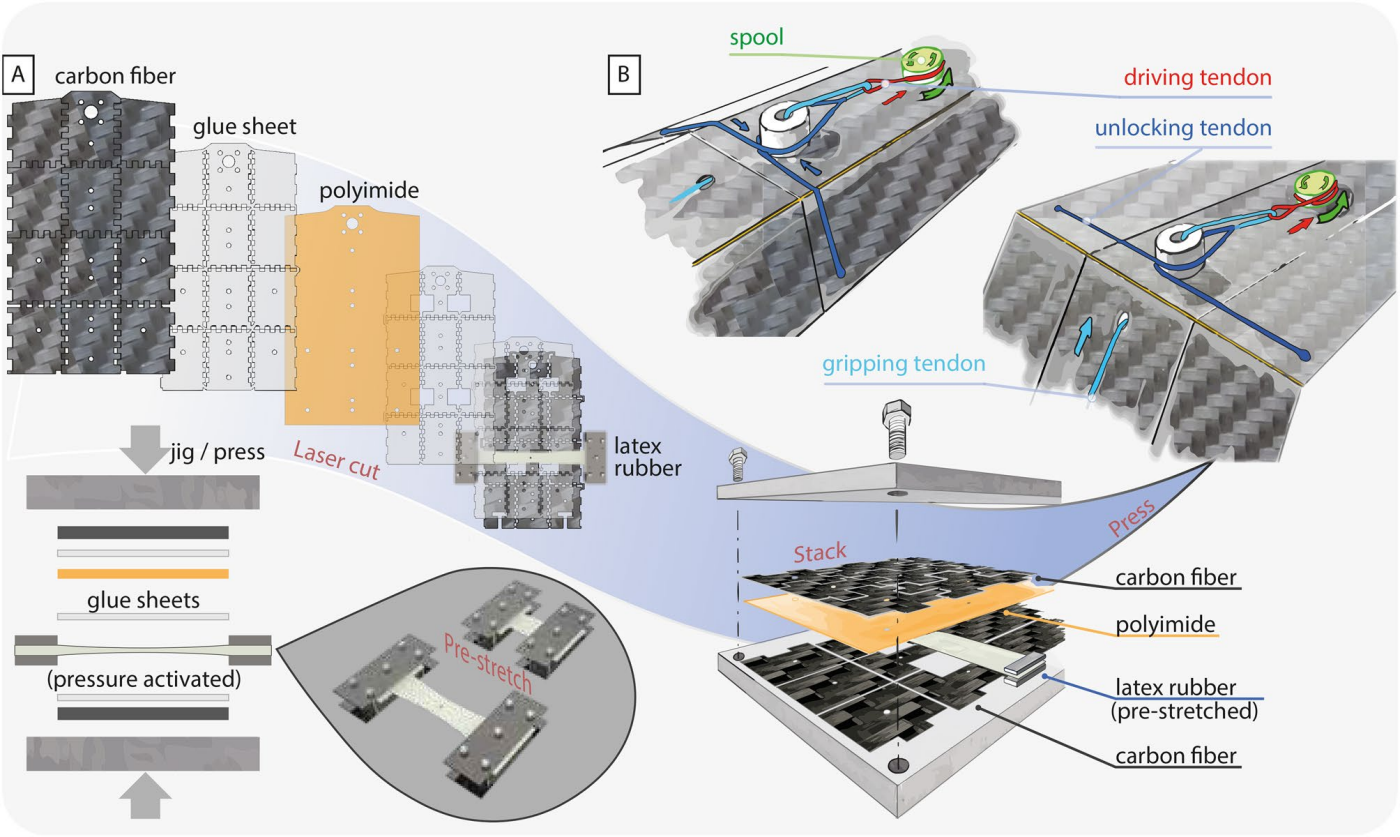
The metamorphic arm. (**A**) The manufacturing process of the morphing arm. The layers are laid on jigs with the elastic membranes pre-stretched before being pressed to form a single panel. (**B**) The actuation of the tri-tendon spool mechanism to unlock the arm. The directions of the tendon retractions are labeled with arrows of their respective color.

The arm utilizes a novel tendon-drive system which occupies minimal space on the platform and can conform to the geometry of the articulated structure (Fig. 2B). Inspired by the sloth’s muscle architecture, the tendon only actuates in the gripping direction (adduction). With the lack of an abductor tendon, the thrust from the rotors is used to return the arm to its rigid configuration. Active actuation in both adduction and abduction means the arm does not require gravity to function. The use of shared actuation and multi-functional components minimizes the weight and complexity of the robot, reducing the energy needed to hold a perch and increasing flight time.

**Figure 3:**
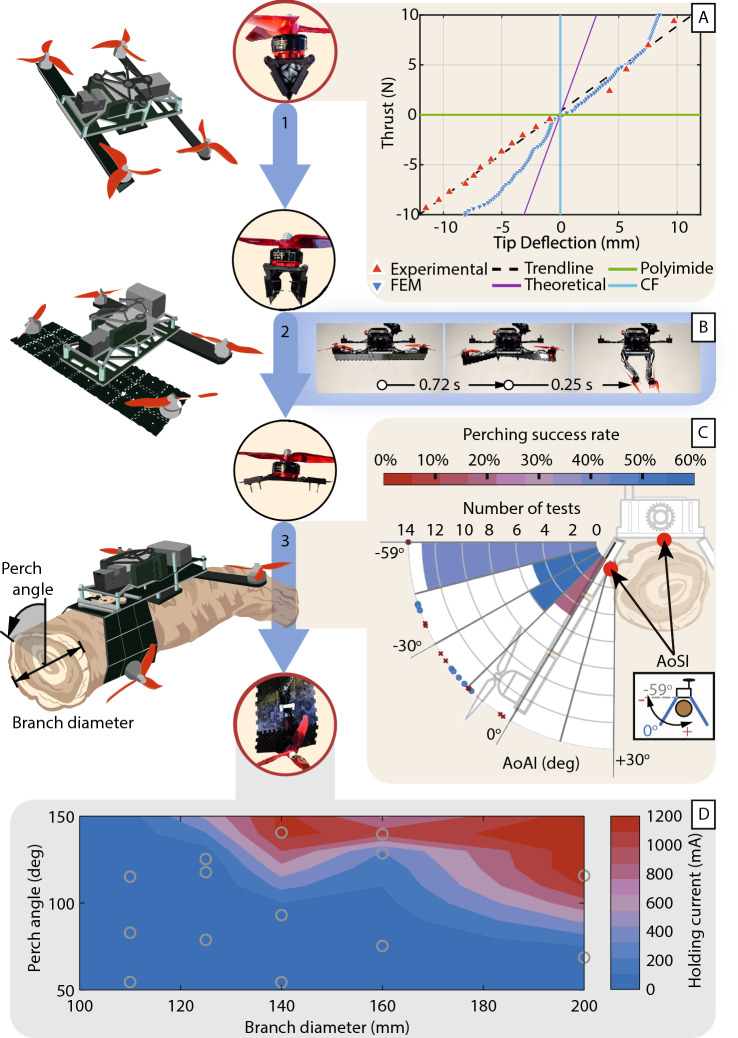
Performance study of the prototype robot from flight to perched. The arm morphing process has three stages: 1. locked to fly, 2. transition to unlocked, and 3. fully unlocked to grasp. The photographs of the arm at each stage are displayed. (**A**) The relationship between the tip deflection and thrust loading, plotted against the analytical models and finite element analysis when the arm is locked. (**B**) The time needed for the arm to open and grasp, determined with a high-speed camera. (**C**) The drop-perch successful rate at the angle of the arm at impact (AoAI), relative to the arm position at the angle of simultaneous arm/body impact (AoSI). A dot indicates a perch success, and crosses are failures. After landing, the robot must grasp the branch (approximated by a cylindrical pipe) to be considered perched. (**D**) The holding current of the servomotor with varying perching angles and branch diameters.

Developed in conjunction with the adaptive bi-stable metamorphic arm is the multi-modal quadrotor robot. The robot transitions between three distinct morphologies: a locked state for flight; an unlocked state with which the arm is fully extended but cannot sustain the forces for flight; a perched state where the robot arm conforms to its target and holds its grasp with minimal energy use (Fig. 1A). The 650g robot with a pair of morphing arms, weighing 48g, demonstrated flight and perching through dynamic and static maneuvers. Our proposed approach of robotic metamorphosis leads to a compact quadrotor design that autonomously reconfigures within 0.97 seconds between flying and perching (Fig. 3B).

## Results

### Morphing robot arm design

The robot arm has three morphological states: triangular, flat, and curled (Fig. 1A). In these respective states, the robot can lock its arm for flight, transition between triangular and curled configurations, and grip onto tree branches to perch. The robot arm is formed as a carbon fiber-polyimide-carbon fiber sandwich panel (Fig. 2A), a technology inspired by other lightweight articulated robots to create lightweight hinge joints^69,70^. The layers are bonded using pressure-activated glue sheets. The DuPont$^{\hbox {TM}}$ Kapton®HN polyimide layer acts as hinges, allowing the carbon (fiber) panels to have articulated movement. From the arm’s root to its tip, it has two rows of axial hinges, enabling the arm to fold into an open-section triangular cylinder. The arm can have an arbitrary number of rows of lateral hinges across the arm. The arm rotates about its lateral hinges to grip and perch. Elastic membranes, embedded in the sandwich panel, hold the arm in the triangular state. The membranes are pre-stretched prior to layer stacking and pressing. The pre-stretch is accounted for when the membranes are cut. The sacrificial tabs ensure a repeatable pre-stretch and are removed during final assembly. The membrane is offset from the center of the polyimide hinges to ensure that there is a moment arm for the elastic membrane to bend the arm to its natural triangular state. Overlapping teeth are cut on the outer carbon fiber sheet to act as mechanical endstops, restricting the arm’s motion to its designed workspace. Steel spines are embedded into the arm’s tip panels to improve perching performance (Fig. 1C).

**Figure 4:**
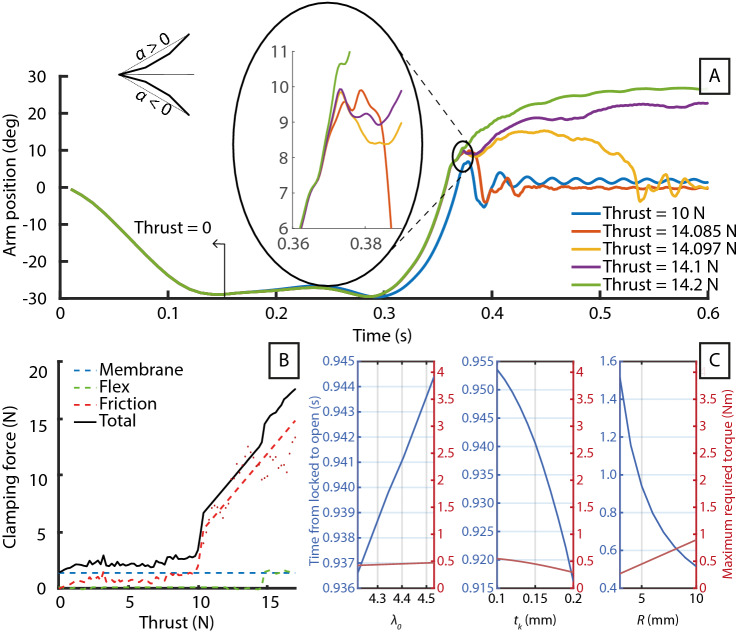
Simulation data. (**A**) Dynamic finite element simulation of the effect of thrust on the morphing arm’s locking behavior and (**B**) the clamping force holding the arm in the locked state. (**C**) Parametric studies of how the arm geometries affect the locked-to-unlocked transition time and servomotor torque requirement using numerical simulation.

The interlocking teeth on the edge of the sandwich panel exhibit a self-locking behavior when the arm is locked. As thrust is applied, the strain of the panels forces the teeth at the two open sides to brace on each other. Finite Element Analysis (FEA) shows that the clamping force between the teeth increases with the thrust load (Fig. 4B). This clamping force enables the triangular cross-section to hold its shape, thus resist both upward and downward loads on the thrust axis. The stiffness of the locked arm is modeled analytically with Euler-Bernoulli beam theory, modeled numerically with FEA, and experimentally verified with a load test in both upward and downward directions (Fig. 3A). The results show that the analytical multi-material Euler-Bernoulli beam model is stiffer than the physical beam, but is within the order of magnitude.

The mismatch of analytical and experimental results is expected as a number of phenomena, not modeled, could have reduced the arm’s stiffness. We align the CF sheets at $0/90^\circ$ fiber orientations along the arm during manufacturing and used the corresponding tensile modulus in the theoretical calculations. But even in the least stiff fiber orientation scenario, $\pm 45^\circ$, the analytical model still differs vastly from the numerical and experimental results. Due to the difference in tensile modulus between the CF sheets and the polyimide sections, the polyimide sections play a much larger role in the arm stiffness, and the geometrical changes to the arm’s cross section at high displacements greatly affected the stiffness of the overall structure. The Euler-Bernoulli model does not account for the glue sheets—it deforms plastically, has a low stiffness, and will shear within the sandwich, allowing the polyimide sheet to shift within the sandwich. This is hinted at by the tip deflection hysteresis shown when load cycling on the prototype arm (see Supplementary Fig. S2). Also, the geometrical changes to the arm cross section at high displacements greatly affected the stiffness of the overall structure. We can only conclude that Euler-Bernoulli beam theory is insufficient to accurately model the arm during bending. However, the FEA result closely matches the experimental data on the upward thrust load until 7*N*, but is overly stiff when under downward thrust.

### Tendon drive design

The perching action is controlled by a servomotor-driven tri-tendon system, threaded across the arm and the robot body during assembly. The driving tendon is wound around the spool and transmits the tension to the T-tendons on both arms. The T-tendon comprises an unlocking tendon, running across the width of arm, and the gripping tendon, threaded from the tip to the root of the arm (Fig. 2B).

The servomotor turns the spool to actuate the arm. This retracts the driving tendon which, in turn, pulls on the T-tendons. As the arm is still in the locked state, the gripping tendon cannot move; thus, the unlocking tendon is retracted towards the spool, opening the arm. The T-tendon acts as a differential, diverting the tension from the driving tendon between the unlocking and gripping tendons. Once the unlocking tendon has reached its limit, the arm is opened. Continuing the retraction of the driving tendon past this limit will begin the pulling of the gripping tendon, contracting the arm and perching the robot. The arm’s unlocking time is entirely dependent on intrinsic design geometries, such as the speed and torque of the servomotor, the spool radius *R*, the thickness of the polyimide joints $t_k$, and the pre-stretch ratio of the latex rubber between assembly and natural lengths $\lambda _0$. Therefore, the design of the morphing arm is guided by numerical models (Fig. 4C). The unlocking time decreases exponentially and torque increases linearly to the increase of the spool radius *R*. As the model does not indicate any maxima or minima, we compromise between the servomotor weight, torque, and speed and the spool’s strength requirements set the actual spool radius at 5 mm.

We designed the self-locking feature to reduce the load on the servomotor as the arm transitions towards the curled state. When spooling in the driving tendon, the unlocking tendon is acting against the elastic membranes to open the arm. The force from the membrane is mostly exhibited as a moment about the axial joint. However, when the arm is in the flat state, the force from the membranes predominately becomes a compression perpendicular across the axial joint. There remains a bending moment due to the offset of the membrane from the hinge axis. The curling of the arm creates an interference between the rows of panels; the bending moment becomes a compressive force on the subsequent row of panels. In other words, once the arm begins the perching motion, a stable equilibrium is reached and no force from the servomotor is needed to keep the arm in the curled configuration. Furthermore, with the quadrotor rotors not producing thrust at perching, the rotors act as weights, increasing the perch speed by overcoming the bending resistance of the polyimide hinges.

When perching, the arm can be considered an underactuated gripper. The sequence of joint movement is thereby dependent on the stiffness of the joints. For perching applications, it is preferable for the gripper to cover the largest possible workspace. The largest workspace is covered by ensuring that the hinges rotate in sequence, starting from the root. In the prototype, the requirements above are met by the embedded variable stiffness mechanism.

The variable stiffness mechanism takes advantage of the thin sandwich panels. With a low cross-section polar moment of area, the panels are prone to twist. By tuning the position where the unlocking tendon attaches to the arm and the location of the elastic membrane(s), the desired moment profile along the axial hinge can be created. For the prototype robot, a low to high hinge stiffness profile, from the arm root to the tip, was chosen. Furthermore, as the lateral hinges progressively bend, it forces the subsequent rows of panels to flatten. This flattening reduces the stiffness of the subsequent hinge, thus reducing the load on the servomotor, increasing the grip speed.

The transition from perched to locked morphology occurs as follows. The servomotor releases the tendon tension. Simultaneously, the rotors generate thrust, providing the force to recover to the flat state. Once in the state, the elastic elements return the arm to the triangular configuration as the spool further unwinds the tendon.

### Modeling of the transition dynamics

The dynamics of the arm, transitioning from perched to locked, are characterized by complex stress states and unstable behaviors. These behaviors are complex to study analytically. Nevertheless, it is crucial to define an operational envelope within which the robot can deperch and return to flight reliably. We chose to analyze the structural dynamics of the arm numerically, the method is detailed in the supplementary material.

There are two distinct behaviors that can occur at the bifurcation point: at low thrusts, the arm folds and regains its stiffness. At high thrusts, this movement proceeds upwards and leads to failure. In practical terms, despite having mechanical limits, when the recovery thrusts to re-lock the arm are too high, the structure can deflect beyond the operational envelope. The bifurcation point of this structural instability is characterized by simulations with iteratively increasing thrust. The declination of the arm’s tip to the horizontal position ($\alpha$) is used as a control variable; a linear scheme finds the next value closest to the instability point. As shown in Fig. 4A, there is no clear boundary defining the two distinct behaviors but instead an instability region with multiple intersecting paths. For the prototype arm, this occurs at thrust values between 14.085 and 14.1 N, which approximately equates to maximum throttle input. However, although $\alpha \rightarrow 0$ is achieved in this region, a violent asymmetric closure of the arm occurs which will likely lead to the misalignment of the overlapping teeth and structural failure. Therefore, a thrust limiter is imposed when transitioning to locked mode.

### Perching mechanics

We studied the baseline performance of the perching mechanism with bench tests. On a test stand, the metamorphic arm transitions from the locked to unlocked state in 0.72 s, and locked to an over $90^\circ$ grasp in 0.25 s (Fig. 3B). During outdoor perching-only tests, we observed that when fully perched upside down the arm would slide slightly until the spines on the arm fully interlock with the tree surface textures (Fig. 1B). Supported by literature^71^, we suspect that friction between the arm and the tree surface is comparatively insignificant to the forces from interlocking. We also noted that, by spreading the steel spines across the three panels on the tip of the arms, it widens the grip—the arm can better resist out-of-plane torques caused by the robot’s center of mass not being located at the root of the arm.

#### Energy usage

The energy cost of perching and flight is determined with onboard current sensors. With a 4-cell lithium-polymer battery and a takeoff weight of 872 g, a typical cruising flight draws an average current of 15A. The robot uses the same battery for perching. The holding current draw depends on the diameter of the branch and the perch angle (Fig. 3D). For smaller branches and perch angles below $60^\circ$ off the vertical, the robot can rest on the branch and only the 40 mA standby current is expended by the servomotor; the gearbox friction is sufficient to hold the perch. At steeper perch angles and with larger branches, the perch holding current increases. At higher holding currents and for extended perch duration, the servomotor will overheat and shut down. This leads to a failed perch. In this event, we can remotely reset the servomotor with an onboard electronic switch.

### Perching flights and strategies

The robot arm reduces its stiffness in the flat and curled state by an order of four magnitudes over its locked configuration. While this increases the compliance of the arm when perching, the arms cannot sustain the thrust load. This results in a highly erratic and uncontrollable flight when transitioning to perch. As the transition process lasts at least 0.72 s (Fig. 3B), we demonstrated two methods to mitigate the inability to fly whiles the arms are not triangular (movie 1).

**Figure 5:**
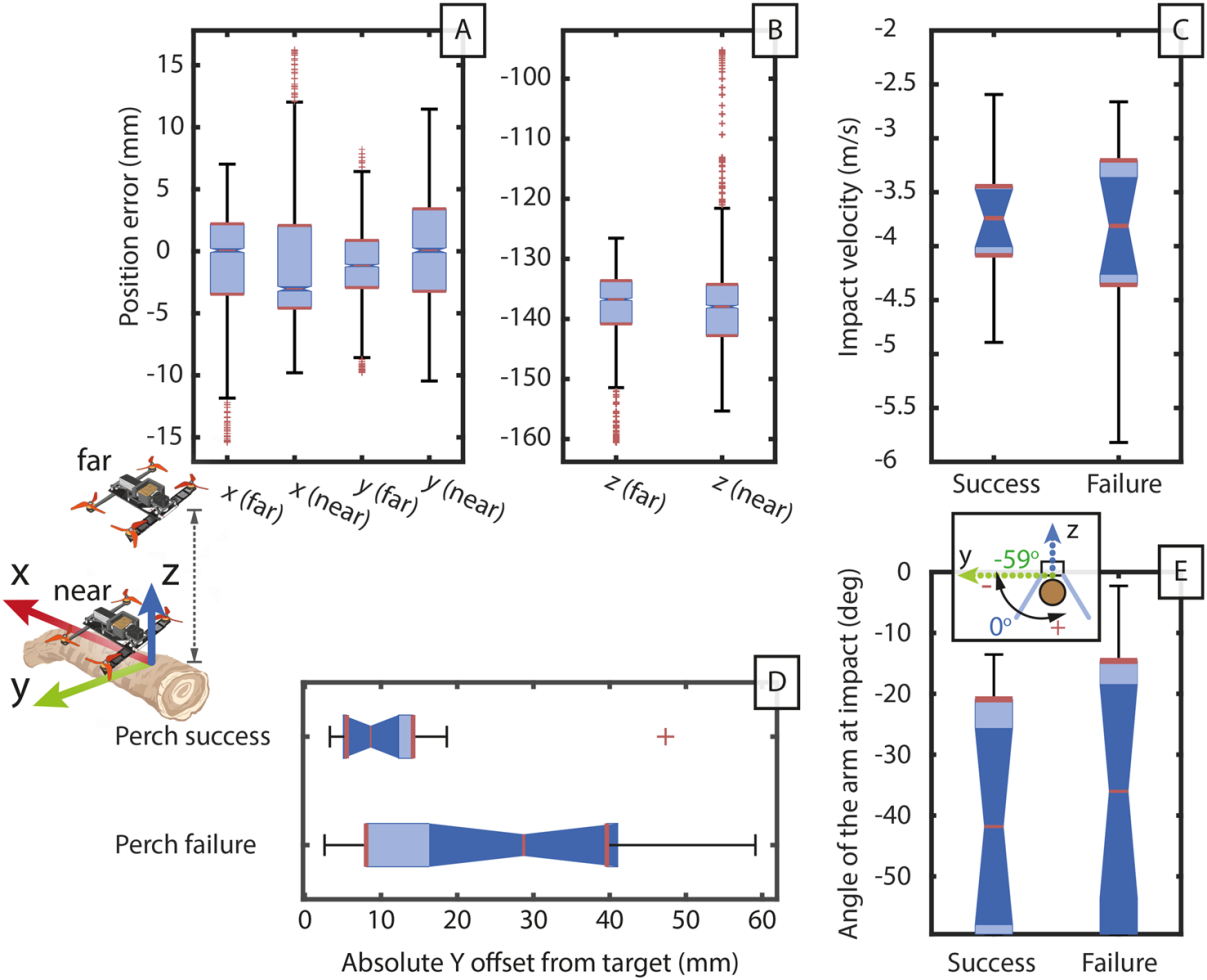
Statistical analysis of the test data. (**A**) Position errors of the robot flying unobstructed and (**B**) near the artificial branch, approximately 1.5*m* and 0.5*m* above the branch, respectively. (**C**) Statistical correlation between perching success/failure and the impact velocity. (**D**) Correlation between perching success/failure and the absolute *Y* offset from the branch at impact. (**E**) Statistical analysis of the drop-perch data.

The first method, which we call “land-perch”, ensures that the robot spends the minimum time in the un-powered and un-perched state, thereby reducing the likelihood of failing to perch and falling off the target. The robot can descend gently and maintain a minimal thrust to remain level in the locked configuration whiles resting on the target. The rotors the throttle down at the beginning of the transition to perch from the unlocked state. However, flying close to the target branch causes significant turbulence, resulting in substantial difficulties aligning the robot to the branch in horizontal directions. This is evidenced by the higher *x* and *y* error interquartile range when flying near the branch (Fig. 5A and B).

Alternatively, the robot can hover high above the target and perform an un-powered drop onto it to minimize the aerodynamic disturbance. We call this method the “drop-perch”. The drone will cut power to the rotors and begin the perching motion while in mid-air. The throttle cutoff is synchronized to ensure that the free-falling robot impacts the target at the perched state. This method is more demanding of the controller’s positional precision. But as the robot can hover with minimal turbulence and is not at risk of collisions, drop-perch avoids the complex interactions of the land-perch. This ability can be further expanded to allow throttle cutoff when the robot launches itself onto a ballistic trajectory to enable perching on inclined structures.

#### Land-perching

**Figure 6:**
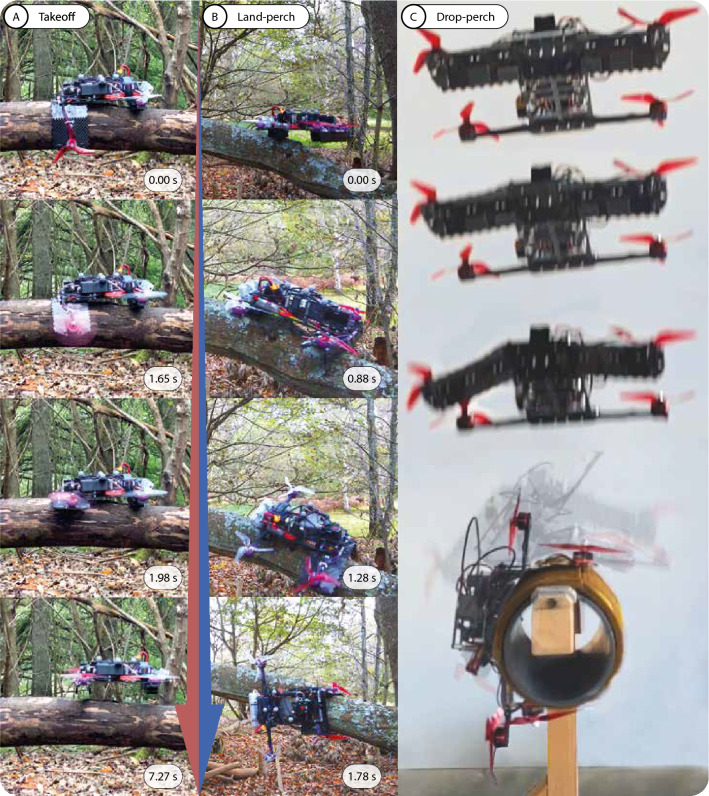
The morphing robot perching indoors and outdoors. (**A**) The robot manually taking off from a perch on a smooth bark tree. (**B**) The robot performing a manual land-perch. (**C**) The robot performing an autonomous drop-perch use motion capture odometry. The robot bounced slightly upon impact, it then firmly gripped onto the side of the fibrous pad covered artificial branch, diameter 140 mm.

The land-perch method is demonstrated both indoors (movie 2), with motion capture, and manually outdoors (movie 3). The transition from perch to flight is also validated from a static perch (Fig. 6A).

**Figure 7:**
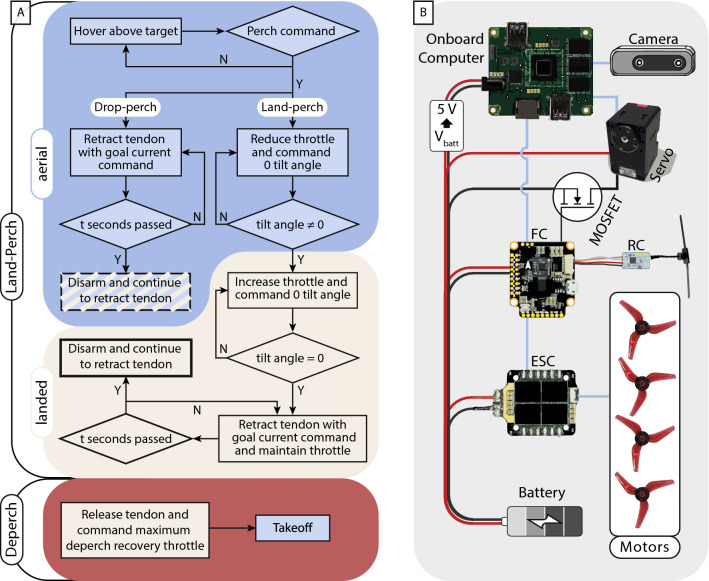
The control flow chart and electronics system. (**A**) The finite state machine of the land-perch procedure, the drop-perch controller, and the deperch process. (**B**) The electronics architecture of the robot with an additional N-channel MOSFET to switch on and off the servomotor.

The indoor land-perch method utilized a finite state machine to detect the contact with the target branch. The detection is determined by the mismatch between the throttle and the vertical acceleration. Upon detecting the contact, the robot attempts to remain balanced on the structure with minimal throttle. This gives the arm sufficient time to morph and grip before all rotors shut off in a successful perch. These three distinct behaviors are encoded with the logic presented in Fig. 7A.

While the indoor land-perch method relies entirely on the robot’s onboard sensors, the positioning of the robot prior to perching utilizes motion capture. The limitations of the onboard tracking camera in forest environments restricted the outdoor tests to manual piloting. Nevertheless, the robot can perch on an inclined branch (Fig. 6B). The smoothness of the branch and the slight position offset from the branch center caused the robot to slide off when the motors were shut off. However, the arms continued to grip and perched the robot at approximately $90^\circ$ from the horizon.

#### Drop-perching

We first tested the ability of the robot to drop-perch with an electronically timed-release mechanism. The drop-perch is then demonstrated during autonomous flights in an indoor environment (movie 4). To better control the size of the branch, the drop-perch is tested with an artificial branch made with the method described in Experimental Setup. While it is too inaccurate to quantitatively extrapolate the perching performance from the artificial branch to trees in the rainforests, the photographs shown in Figs. 1B and 6B of the robot perching on a coarse and smooth branch, respectively, indicate that the robot is likely capable of perching on the smooth barks of tropical rainforest trees^72^.

We observed two distinct drop-perching mechanics. If the body makes initial contact with the branch at impact, the front of the drone would bounce. The morphing arm, at the back of the robot, would remain in contact with the branch and grip. The arm grips firmly onto the branch at the rebound of the front body. Alternatively, if the arms are positioned such that they make contact first, they would deform to dampen the impact. Providing that the arms deform outwards such that it exposes the spines, the robot perches without rebounding.

However, if the arms contract too much at impact, the impact causes the arms to deform inwards towards the body. This morphology hides the spines and results in a failed perch. Another failure mode is the arms not gripping in time to prevent the robot from sliding off the branch. This failure mode is easily remedied by increasing the height of the drop, allowing the arm the time to contract more. At high impact speed and energy, the robot will rebound off the branch. But upon the second impact, if the branch is still within the range of the arm, the robot successfully perches (Fig. 6C).

An intentionally unoptimized flight controller tuning created a natural random scatter in the drop-perch data (Fig. 3C). While this affected the drop-perch success rate, this scatter allowed us to capture the breadth of perching behaviors across varying impact velocities (Fig. 5C), sideways *y* offset (Fig. 5D), and the angle of the arms at impact (AoAI) (Fig. 5E). The AoAI is determined relative to the angle of simultaneous arm/body impact (AoSI), approximately $-59^\circ$ from the horizontal during a vertical drop. The data suggests that the optimum arm angle at impact is between $-27^\circ$ and $-58^\circ$ from the AoSI on a 140 mm diameter branch. Interestingly, the AoAI for failed drop-perches are closer to AoSI than for successes, this is evident with the difference in mean and interquartile range. Therefore, we theorize that a slightly wider arm position increases the robustness to off-centered impacts without causing too much detriment to the gripping speed.

### Vehicle mass breakdown

The integrated metamorphic design allows components on the robot to perform multiple roles, reducing the overall weight. For the aerial robot, the rotors on the perching arm are used to recover the arm when transitioning to flight. Not accounting for the battery mass but including the flight computer, the flight and perch subsystems weigh 430 g and 383 g respectively. As an integrated subsystem, the flight and perch subsystem weighs 541 g, giving a shared mass of 272 g. The shared mass constitutes $49 \%$ of the integrated subsystem; a $33 \%$ mass reduction from modular design, where the subsystem weights are summed, to integrated design. The component mass breakdown is given in Supplementary Table S2.

## Discussion

In utilizing metamorphic designs on small multirotor UAVs, we have created a monolithic robot that can fly and perch. The integration and sharing of components on the flight and perching subsystems resulted in a 208 g mass reduction on the 650 g robot. Using the full span of the multirotor structure to perch, the robot can grip onto tree branches of various geometries, diameters, and in various angles. We demonstrated this with handheld tests on fibrous pad-covered pipes and perching flight tests with a sectioned tree branch. The proposed perching methods, land-perch and drop-perch, were demonstrated autonomously in an indoor environment with motion capture. In addition, the land-perch was demonstrated manually in a forested environment. Having the gripper integrated into the robot body lowers the centroid of the robot to 18 mm above the base of the arm at perch. The robot’s low center of gravity reduces the moment arm when the robot’s center of mass is offset from the center of the branch, reducing the peeling moment and lowering the holding torque required from the perching mechanism.

While substantial weight is saved by using integrated metamorphosis, there are penalties to consider. Most significantly, in making the quadrotor arm articulated, the structure is weaker at the hinges. Furthermore, the metamorphic perching transition process and the use of an underactuated gripper configuration result in complex system dynamics. While we mitigated this issue by perching and taking off rapidly, the underlying control problem remains unsolved.

For the robot to function autonomously outdoors, a few improvements must be made. When the robot slips during gripping, it would perch at an acute angle (Fig. 6B). While this does not affect the perching performance or its ability to transition back to the triangular configuration, taking off from this position requires the robot to fall a substantial distance when self-righting. This can be mitigated by aborting the perch when the onboard sensors measure a large angular velocity after detecting contact with the branch, thereby reducing the transition time to flight as the arm would not be fully contracted.

Identification of suitable perch locations is also an intriguing challenge. As shown in Fig. 6B, the robot can land-perch on inclined branches. But the maximum incline, suitable geometries, and other limiting branch characteristics remain unknown. Current perch locations are determined by trial and error. Identifying suitable locations prior to perching would be a major advancement towards full automation.

Integrated metamorphosis is a design principle that can be further explored in numerous ways^73^, some of which have already been shown in other types of robots with capabilities such as self-assembly^74^. Our design is also not limited to a metamorphic arm. Adding the bi-stable metamorphic mechanism to the body of the drone could increase its compliance to aid perching on vertical branches, similar to a gliding gecko^75^. The large surface area of the arm in its flat state can be utilized as an aerodynamic surface for gliding flight. Many situational behaviors were also left unexplored. An example of such is our current interest; a method of low-power aquatic locomotion, whereby the robot would perch on floating objects to maintain buoyancy and steer with the rotors on the rigid arms.

To conclude, the multi-modal capability gained through integrated metamorphosis, in conjunction with inspiration from tree-dwelling animals, enables us to imagine synergetic mission profiles where robots can spend a greater proportion of the operation at rest than in flight. Our results have displayed the wealth of potential capabilities that can be gained by applying metamorphic designs, producing compact robots that are suitable for the autonomous exploration of the natural world.

## Methods

### Robot construction

The metamorphic arm is a sandwich of 0.5 mm carbon fiber reinforced polymer panels, 0.075 mm polyimide sheets, and elastic latex membranes bonded with acrylic glue sheets (Fig. 2). The carbon fiber panels are pre-cut on an Oxford laser, and the others on a Universal laser. We align the sandwich with pins on a jig and then we pressed the sandwich to activate the pressure-sensitive glue sheet. Tabs on the carbon fiber panels are then removed, allowing the arm to fold. Two arms are interlinked at manufacturing to form an arm pair. This offered increased stiffness during flight and improved the synchronization of the arms during perching. Steel spines are glued onto the arm at an angle similar to perching animal claws (Fig. 1C)^76^. The stainless-steel servomotor spool is laser sintered. The mounting brackets for the servomotor and tracking markers, and the flight computer and camera are fuse-deposition manufactured with polylactic acid (PLA) and thermoplastic polyurethane (TPU) respectively.

The robot body uses a twin deck 3 mm carbon fiber plate construction with the lower deck providing the mounting for the propulsion components (rotors, electronic speed controllers, flight controller). The upper deck houses the Dynamxiel XH430-W350 smart servomotor, an AAEON Up Core computer, an optional Intel T265 tracking camera, and the battery.

### Control system

Indoor flights were conducted with the robot odometry provided by a VICON motion capture system. For outdoor autonomous flights, the odometry is provided by the tracking camera (Fig. 7B). We implemented a proportional-integral-derivative controller to control the robot’s position and yaw orientation. The control system of the robot is implemented in ROS. The controller outputs commands in the form of roll and pitch angles, yaw angular rate, and thrust percentage. The controller commands along with arm/disarm and the servomotor on/off are sent to the flight controller using MAVROS via a Wifi network. The servomotor is controlled by its proprietary ROS driver. For land-perch and drop-perch, the flight commands and servomotor commands are synchronized using the ROS in-built clock.

### Numerical simulation

The finite element analysis software *Abaqus/Explicit* is used to model a single bi-stable arm. The full dynamic equations of motion are solved explicitly, permitting the solution of large displacements, constitutive nonlinearities, and instability behaviors, all necessary to evaluate the working limits of the mechanism.

The arm is modeled in its planar state using 9 sections of isotropic elastic CFRP-equivalent material connected by hinge sections of polyimide. The pre-stretched latex membrane is modeled using 1D first-order elements and the Mooney-Rivlin hyperelastic constitutive model. As in the physical arm, the central CFRP section of the inner row is constrained in 3 DoF, while all other edges are free to move.

To reduce the computational expense of solving multiple contact pairs, no interlocking teeth are added to the hinge sections. Consequentially, the one-way hinge rotation achieved by the interlocking teeth is not fully captured. We thus use a field variable that switches the section and material properties of the hinges from polyimide to CFRP at the angles that the teeth should interlock, achieving a similar behavior.

The geometry is discretized arranging two-dimensional *S4R* elements with hourglass control and 5 integration points through the thickness in a structured mesh. A structured mesh can be built in this case because the arm is modeled in an initial planar state, which greatly improves mesh convergence. A mesh refinement study was executed for the unfolding step of the simulation using the following variables: first natural frequency after unfolding and the value of the von Mises stress in 2 nodes common to all meshes and located in the polyimide hinges.

Simulations are carried out in multiple steps. For simulations where the stiffness of the arm in flight configuration is to be tested, a first step first allows the arm to passively fold by the action of the pre-stretched membranes. A second simulation step then loads the free end of the arm in the negative and positive direction, and the deflections are measured. For simulations where the instability point on take-off is studied, the inner edge of the arm is initially constrained to allow the arm to pivot towards a perched configuration. This constraint is then removed and a thrust force of varying intensities is applied to the action line of the propulsion system, resulting in the 2 different behaviors demonstrated.

### Tip deflection experimental setup

To determine the tip deflection of the arm when under load, we mounted the arm on a test bench and applied varying weights (Fig. 3A). The arm’s tip deflection is measured with VICON motion capture.

### Perch strength bench test

The required servomotor’s holding current at various perch angles was determined by bench tests. The servo is actuated at maximum current before ramping down until the robot detaches from the pipe. The current is measured by the servomotor’s internal circuitry. The perch strength bench test was attempted with an arm that has steel spines, an arm with sandpaper pads, and one without steel spines or sandpaper.

Under all three setups, the robot was unable to hold onto the smooth PVC artificial branch at a $90^\circ$ perch angle. However, the arm with steel spines is able to hold onto the fiber pad-covered branch at $90^\circ$ while the other two setups failed. We conclude that the effect of interlocking with steel spines is significantly greater than friction when perching on coarse surfaces. Therefore, further testing is conducted using steel spine-embedded arms as they are more applicable for perching on trees.

### Perching flight experimental setup

During the perching tests, the position, velocity, and orientation of the robot, the position of the branch, and the servo position are recorded. For the land-perch, the robot is commanded to slowly descend. Once the robot stably lands on the artificial branch the robot is disarmed while the folding arm grips. For outdoor tests, the robot is manually controlled. When outdoors, the perching process is commanded by a switch on the remote control.

the indoor drop-perching test is carried out by dropping the robot on artificial tree branches, built using round PVC pipes covered by a layer of 10 mm thick non-woven fiber abrasive pads. This setup allows the spines on the arm to grip into the gaps in the fiber pads, mimicking the mechanical interlocking between the spines and the bark of real tree branches. The pipes ranged in diameter from 75 to 200 mm. The artificial branch is marked by tracking markers so that its global position in the motion capture world frame can be obtained. The robot is controlled to fly to the branch position and hover above the branch at a set distance. When the robot hovers stably above the branch, the perching command can be initiated.

When initiating dynamic drop-perch, the goal current commands will be sent to the servo, and the servo will apply a constant grasping force. The arm will start to unfold. A disarm command is then sent to the flight controller after a short delay, timed to maximize control of the robot whiles reducing the duration of free fall. At disarm, the robot loses lift and starts free fall, while the arm continues to unfold and grip. When the robot hits the branch, the arm should be fully unfolded and curled to be ready to grasp. The dropping height and the delay time are crucial for a successful drop-perch. In 33 tests with high-speed camera footage and motion tracking at 90 Hz, the robot executed the free-fall with a mean starting position of +4.4  mm off the center of the branch and a standard deviation of 19.1 mm. Relative to the center of the branch, the mean point of impact is at +3.5 mm with a standard deviation of 25.5 mm; the time of impact is defined as 2 timesteps prior to maximum jerk.

the acceleration is calculated by a forward-difference numerical differentiation of the velocity. We then filter the acceleration with a second-order Butterworth filter at a 40 Hz cut-off frequency. The jerk is then calculated with the filtered acceleration using the forward-difference numerical scheme. A hand-tuned time offset is applied to account for the filter delay. The angle of the arm at impact (AoAI) is calculated by the servo angle, assuming that only the tendon at the root joint is retracted during free fall.

## Supplementary Information

Supplementary Video 1.

Supplementary Video 2.

Supplementary Video 3.

Supplementary Video 4.

Supplementary Information 1.

## Data Availability

The datasets used and/or analyzed during the current study are available from the corresponding author on reasonable request.
