# Supplementary material for: Metamorphic aerial robot capable of mid-air shape morphing for rapid perching
Source: Sci Rep. 2023 Jan 23;13:1297. doi: 10.1038/s41598-022-26066-5 (PMC9870873; doi:10.1038/s41598-022-26066-5)
Supplement: Supplementary file 5 — Supplementary Information 1. [file 41598_2022_26066_MOESM5_ESM.pdf]

The Sloth - Supplementary Material

Peter Zheng, Feng Xiao, Pham Huy Nguyen, Andre Farinha, Mirko Kovac

September 2022

Supplementary Table S(1): **Nomenclature** of the kinematic model

T	Thrust of a single rotor
τ	Servo torque
P	tendon tension
θ	Servo angle of rotation
a	Distance of tendon anchor to the hinge
b	Distance of tendon edge to hinge
α	Internal angle between the links
R	Radius of spool
d	Distance of membrane attachment to the hinge
σ	Stress of elastic membrane
w_m	Width of membrane
t_m	Thickness of membrane
E_k	Elastic modulus of polyimide hinge
L_{arm}	Length of a single arm
t_k	Thickness of polyimide hinge
x_0	Distance between rigid links (hinge gap)
C_{10}	Elastic constant of latex membrane
C_{01}	Elastic constant of latex membrane
λ	Membrane stretch ratio (current/unstretched)
λ_0	Initial stretch ratio (at manufacturing)

1 Deflection of the morphing robotic arm

Approximating the quad-rotor arms as Euler-Bernoulli cantilever beams during flight, we derive vertical deflection z as a function of

$$z(x) = \frac{Tx^2}{2(EI)_{\text{arm}}}(L - \frac{x}{3}) \tag{1}$$

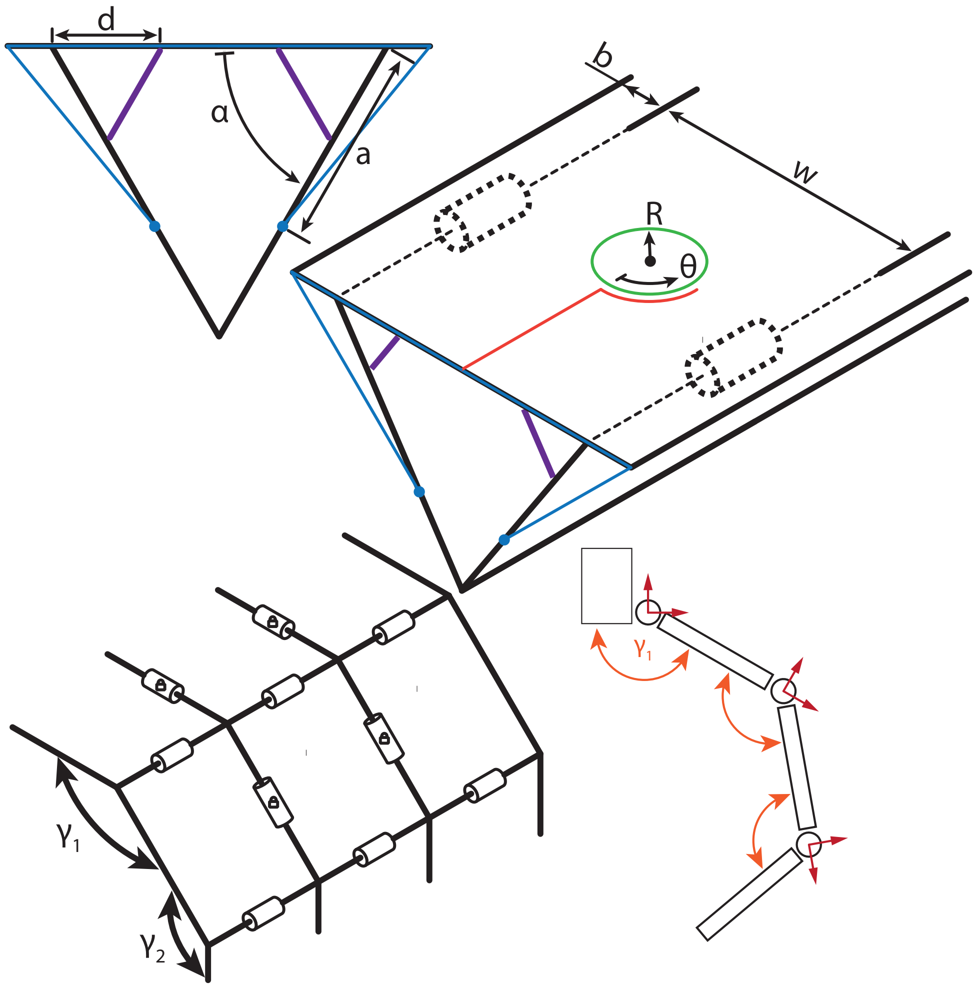

Supplementary Figure S(1): **Diagram of the morphing arm** with the dimensions used to model the structural stiffness and the unlocking dynamics. The cylinders denote the position of the cylindrical polyimide joints.

where T is the thrust of the rotor, L the length of the arm, and the distance from the root of the arm x (Supplementary Table S(1)). The stiffness of the arm $(EI)_{\text{arm}}$, due to the sandwich construction, is bounded by two analytical models. As the arm is comprised of a series of polyimide joints, the lower bound stiffness model assumes that the load is carried by a homogeneous polyimide structure. However, the neutral axis of bending is imposed on the top plane as the carbon fiber plates, magnitudes higher in Young's modulus, will restrict the compression. Further assuming that second order and higher terms of the thickness, t_k and t_{cf}, are insignificant, this gives

$$(EI)_{arm} = 2E_k \int z^2 dA = \frac{E_k t_k w^3}{2} \tag{2}$$

The upper bound model assumes a homogeneous carbon fiber structure. With the neutral axis of bending imposed at the centroid of the cross section, this gives

$$(EI)_{arm} = E_{cf} t_{cf} w^3 \left(\frac{23}{8} - \sqrt{3} \right) \tag{3}$$

We can further model the arm as connected homogeneous sections, alternating between polyimide and carbon fiber, giving equations in the form

$$\frac{dz}{dx}|_{i<x\leq i+1} = \frac{T}{(EI)_i} \left((x - X_i) l_i - \frac{1}{2}(x - X_i)^2 \right) - \frac{T(X_i - L)}{(EI)_i}(x - X_i) + \frac{dz}{dx}|_{x=i} \tag{4}$$

$$z_{i<x\leq i+1} = \frac{T}{(EI)_i} \left(\frac{l_i}{2}(x - X_i)^2 - \frac{1}{6}(x - X_i)^3 \right) - \frac{T(X_i - L)}{2(EI)_i}(x - X_i)^2 + \frac{dz}{dx}|_{x=i}(x - X_i) + z_i \tag{5}$$

with initial conditions

$$\frac{dz}{dx}|_{x=0} = 0 \quad , \quad z_0 = 0 \tag{6}$$

and $X_i = \sum_0^i l_i$.

2 Parametric numerical study of the tendon actuation

The transition dynamics from the locked to unlocked state is determined by the characteristics of the servomotor-spool, the elastic elements, the polyimide hinges, and the tendon anchor position (Supplementary Figure S(1)).

The servomotor is modelled as a DC motor with a linear speed-torque curve such that

$$\tau = \tau_{\text{stall}} \left(1 - \frac{\text{RPM}}{\text{RPM}_{\text{no-load}}} \right) \tag{7}$$

$$\dot{\theta} = \frac{2\pi}{60} \text{RPM}_{\text{no-load}} \left(1 - \frac{\tau}{\tau_{\text{stall}}} \right) \tag{8}$$

The speed of the arm unfolding is estimated by a forward euler numerical scheme with the servo angular rate ($\dot{\theta}$) determined by the torque (τ) at each time step.

Using a Mooney–Rivlin model, assuming the membrane to be incompressible (Poisson ratio= 0.5), the stress of the membrane, σ is

$$\sigma = \frac{2}{\lambda}\Big(C_{10} + \frac{C_{01}}{\lambda}\Big)\Big(\lambda^2 - \frac{1}{\lambda}\Big) \tag{9}$$

with the stretch ratio (λ) defined as the length ratio of the stretched and unstretched membrane length. Thus

$$\lambda = \frac{l_{\text{stretched}}}{l_{\text{unstretched}}} = \frac{l_{\text{st.}}}{l_0}\frac{l_0}{l_{\text{unst.}}} = \frac{l_{\text{st.}}}{l_0}\lambda_0 \tag{10}$$

which for the geometry of the prototype

$$\lambda = \lambda_0 \sin\frac{\alpha}{2} \tag{11}$$

The moment due to the stiffness of the polyimide hinge M_{joint} is modelled as an Euler-Bernoulli beam of uniform curvature

$$\epsilon = \frac{w_k - w_{k0}}{w_{k0}} = \frac{(r+t)(\pi-\alpha) - r(\pi-\alpha)}{w_{k0}} = \frac{t(\pi-\alpha)}{w_{k0}} \tag{12}$$

$$M_{\text{joint}} = \int_{-\frac{t_k}{2}}^{\frac{t_k}{2}} E_k \epsilon t L_{\text{arm}} dt \tag{13}$$

$$= \frac{E_k L_{\text{arm}}(\pi-\alpha)}{w_{k0}} \int_{-\frac{t_k}{2}}^{\frac{t_k}{2}} t^2 dt \tag{14}$$

$$M_{\text{joint}} = \frac{E_k L_{\text{arm}}(\pi-\alpha)t_k^3}{12 w_{k0}} \tag{15}$$

where ϵ is the strain The moment equilibrium at the hinge gives

$$F_t a \sin\beta + \frac{E_k L_{\text{arm}}(\pi-\alpha)t_k^3}{12 w_{k0}} = F_m d \cos\frac{\alpha}{2} \tag{16}$$

$$\frac{F_t ab \sin\alpha}{\sqrt{a^2+b^2+2ab\cos\alpha}} + \frac{E_k L_{\text{arm}}(\pi-\alpha)t_k^3}{12 w_{k0}} = F_m d \cos\frac{\alpha}{2} \tag{17}$$

assuming frictionless tendon motion. F_t denotes the tension of the tendon, the force of the membrane $F_m = \sigma w_m t_m$, β the angle between the tendon and the side links of the arm, and m the length of the tendon between the side links and the tendon pivot.

The angle between the sections of the arm (α) is formulated as a function of the servo angle (θ),

$$\alpha(\theta) = \cos^{-1}\Big(\frac{R^2\theta^2 + ab - R\theta\sqrt{a^2+b^2+ab}}{2ab}\Big) \tag{18}$$

where $\theta = 0$ is the neutral state of the arm in flight configuration.

The tension of the tendon is further multiplied eightfold by the number of actuated tendons on an arm pair and the pulley system. The torque required is formulated as a function of the servo angle (θ) (see Fig.2)

$$\begin{aligned}\tau(\theta) =& \frac{8R\sqrt{a^2 + b^2 + 2ab\cos\alpha}}{ab\sin\alpha} \\ & \cdot \left(\sigma w_m t_m d \cos\frac{\alpha}{2} - \frac{E_k L_{\text{arm}}(\pi - \alpha) t_k^3}{12 w_{k0}}\right)\end{aligned} \tag{19}$$

$$\text{At } \alpha = \pi, \qquad T(\theta_{\alpha=\pi}) = \frac{R d A_m (a - b)\sigma(\pi)}{2ab} \neq 0 \tag{20}$$

where definitions are given in table S(1). Finally, (19) can be substituted into (8) and numerically advanced in time to determine the position of the arm.

A parametric study was conducted to determine the trade-offs between actuation speed and servomotor limits. Predictably, the opening time increased with increasing membrane thickness and stretch, and decreased with increasing hinge thickness. However, the servomotor's torque-speed characteristics and spool manufacturing constraints result in an optimum spool radius limited by hardware.

3 Video analysis

The transition times between the three configurations on the prototype robots are determine by video analysis. During this analysis, the robots are rigidly mounted to a test frame. While the folding and unfolding speeds vary due to the variances in the manufacturing, the robots are able to achieve locked-to-unlocked times of less than $1s$. A further $0.25s$ is required for both the unlocked arms to sweep pass $\pi/2$.

The flight behaviours of the robot during perching transition was further examined with high speed video. The high speed video was captured with an Apple iPhone 11 Pro. The platform, while in a semi-flexible state in mid-transition, was sufficiently rigid to exert some stabilizing forces. Thus, the perching transition can begin prior to motor cutoff, reducing the glide time.

4 Robot mass breakdown

The mass breakdown of the prototype robot is as shown in Supplementary Table S(2).

5 Tip deflection data analysis

The deflection of the arm tip is tested using weights hung from the arm tip. This test approximates thrust loading from the rotor. The load is fully taken off the arm each time before a new

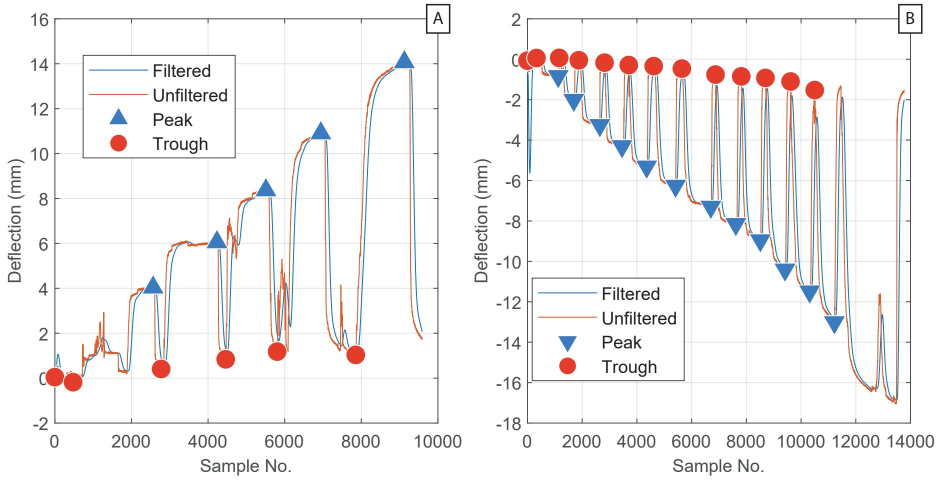

Supplementary Figure S(2): **Arm tip deflection experimental data** using motion tracking of the arm on a test bench where a static load is applied. (**A**) When a positive thrust load is applied. (**B**) When a negative thrust load is applied.

load is applied (Fig. S(2)). The weight was gently applied to ensure no effects from momentum. To denoise the data from motion capture, the z displacement was low pass filtered. A sliding window peak and trough finding algorithm finds the deflection at zero load and full load at each load cycle.

6 Flight testing and data analysis

The T265 camera was mounted on the platform during all perching tests. However, during outdoor testing using the camera's visual inertial odometry, we found that the VIO output drifts significantly in the absence of contrasting and angular objects. This issue was confirmed qualitatively with flight tests in four settings; indoor, forest understory, grass land, grass land with a scattering of man-made objects.

For statistical analysis of the drop-perch success rate, the arm is assumed to be straight at the moment of impact. Thus, we can estimate the arm position, defined by the angle of the root joint, from the servomotor position. The MATLAB script is provided below.

```
%% Sloth drop test script
% find all available data
folders=dir('official_test');
folders(~cell2mat(extractfield(folders,'isdir')))=[];
n_folder=size(folders,1);

% extract relevant data from the folders
```

```
if ~exist('Data_processed')
    k=3;
    for i=4:n_folder
        dum_name =join([folders(i).folder,'\',folders(i).name,'
           \_slash_Flight_Data.csv']);
        dum_name1=join([folders(i).folder,'\',folders(i).name,'
           \_slash_mavros_slash_imu_slash_data.csv']);
        dum_name2=join([folders(i).folder,'\',folders(i).name,'
           \_slash_dynamixel_workbench_slash_dynamixel_state.
           csv']);
        dum_name3=join([folders(i).folder,'\',folders(i).name,'
           \_slash_vicon_slash_pipe_slash_pose.csv']);
        if exist(dum_name)
            %          if exist(dum_name) && contains(folders(i)
               .name,'05-05') && ~contains(folders(i).name
               ,'05-05-14')
            Data_raw(i-k).name=folders(i).name;
            Data_raw(i-k).FD=readtable(dum_name);
            Data_raw(i-k).IMU=readtable(dum_name1);
            Data_raw(i-k).Ser=readtable(dum_name2);
            Data_raw(i-k).Ser.Properties.VariableNames = {'
               rosbagTimestamp' 'Dynamixel' 'Name' 'ID' '
               Position' 'Velocity' 'Current'};

            if exist(dum_name3)
                Data_raw(i-k).Pipe=readtable(dum_name3);
                pipe_height(i-k)=Data_raw(i-k).Pipe.z(1);
            else
                Data_raw(i-k).Pipe=0;%Data_raw(i-k-1).Pipe;
                Data_raw(i-k).Note='no pipe pose';
            end

        else
            k=k+1;
        end
    end
    mean_pipe_height=sum(pipe_height)/sum(pipe_height~=0);
    %          end
    clear n_folder folders dum_name i
    n_data=size(Data_raw,2);
    disp('Data acquired')
```

```
else
    disp('Skip data acquisition')
end

if ~exist('processed')||~processed
    Data=Data_raw; % create a copy, DO NOT CLEAR Data_raw OR k,
        else data acquisition will run.
    wc=40; % butterworth cutoff freq

    for i=1:n_data

        Data(i).t_raw=Data(i).FD.secs+Data(i).FD.nsecs*1e-9;
        % using flight data time as clock
        Data(i).dt=Data(i).t_raw(2:end)-Data(i).t_raw(1:end-1);
        Data(i).Dt=mean(Data(i).dt); % find mean timestep

        index_t0=find(all(cell2mat(Data(i).FD.Status)=='"
           Disarmed"',2),1,'first');  % find the index of first
            "Disarmed"
        if isempty(index_t0) % if cannot find index of first "
           Disarmed", use the start of servo movement
            index_t0=find(Data(i).Ser.Velocity~=0,1,'first');
            t_imu=Data(i).IMU.secs+Data(i).IMU.nsecs*1e-9-Data(
               i).Ser.rosbagTimestamp(index_t0)*1e-9;
            t_servo=Data(i).Ser.rosbagTimestamp*1e-9-Data(i).
               Ser.rosbagTimestamp(index_t0)*1e-9;
            t_pipe=Data(i).Pipe.secs+Data(i).Pipe.nsecs*1e-9-
               Data(i).Ser.rosbagTimestamp(index_t0)*1e-9;
            t=Data(i).t_raw-Data(i).Ser.rosbagTimestamp(
               index_t0)*1e-9;
            Data(i).t=Data(i).Dt*((0:length(Data(i).FD.
               rosbagTimestamp)-1)-find(min(abs(t))))'; % align
                to the closest to zero
        else
            t_imu=Data(i).IMU.secs+Data(i).IMU.nsecs*1e-9-Data(
               i).t_raw(index_t0);
            t_servo=Data(i).Ser.rosbagTimestamp*1e-9-Data(i).
               t_raw(index_t0);
            t=Data(i).t_raw-Data(i).t_raw(index_t0);
            if size(Data(i).Pipe,1)~=1
                t_pipe=Data(i).Pipe.secs+Data(i).Pipe.nsecs*1e
```

```
            -9-Data(i).t_raw(index_t0);
    end
    Data(i).t=Data(i).Dt*((0:length(Data(i).FD.
       rosbagTimestamp)-1)-index_t0)';
end
Data(i).delay=str2num(Data(i).name(6:9));

%% interpolate position and velocity with mean timestep
Data(i).x=interp1(t,Data(i).FD.x,Data(i).t);
Data(i).y=interp1(t,Data(i).FD.y,Data(i).t);
Data(i).z=interp1(t,Data(i).FD.z,Data(i).t);
%          Data(i).y_cmd=interp1(t,Data(i).FD.y_command,
   Data(i).t);
Data(i).vx=interp1(t,Data(i).FD.vx,Data(i).t);
Data(i).vy=interp1(t,Data(i).FD.vy,Data(i).t);
Data(i).vz=interp1(t,Data(i).FD.vz,Data(i).t);
Data(i).ax_imu=interp1(t_imu,Data(i).IMU.x_2,Data(i).t)
   ;
Data(i).ay_imu=interp1(t_imu,Data(i).IMU.y_2,Data(i).t)
   ;
Data(i).az_imu=interp1(t_imu,Data(i).IMU.z_2,Data(i).t)
   -9.80665;
Data(i).servo_pos=interp1(t_servo,Data(i).Ser.Position,
   Data(i).t);
Data(i).servo_vel=interp1(t_servo,Data(i).Ser.Velocity,
   Data(i).t);
Data(i).servo_amp=interp1(t_servo,Data(i).Ser.Current,
   Data(i).t)*2.69; % times by 2.69mA/step https://
   robotis.co.uk/robotis-xm430-w350-t.html
if size(Data(i).Pipe,1)==1 % check height of the table
   to see if pipe position was recorded
    Data(i).pipe_x=ones(length(Data(i).t),1)*Data(i).FD
       .x_command(index_t0);
    Data(i).pipe_y=ones(length(Data(i).t),1)*Data(i).FD
       .y_command(index_t0);
    Data(i).pipe_z=ones(length(Data(i).t),1)*
       mean_pipe_height;
else
    Data(i).pipe_x=interp1(t_pipe,Data(i).Pipe.x,Data(i
       ).t);
    Data(i).pipe_y=interp1(t_pipe,Data(i).Pipe.y,Data(i
```

```
        ).t);
    Data(i).pipe_z=interp1(t_pipe,Data(i).Pipe.z,Data(i
        ).t);
end


%% get initial position of the servo
Data(i).servo_init=Data_raw(i).Ser.Position(1);
%% remove NaN at start and end
while(isnan(Data(i).x(1))||isnan(Data(i).y(1))||isnan(
   Data(i).z(1)) ...
        ||isnan(Data(i).vx(1))||isnan(Data(i).vy(1))||
           isnan(Data(i).vz(1)) ...
        ||isnan(Data(i).ax_imu(1))||isnan(Data(i).
           ay_imu(1))||isnan(Data(i).az_imu(1)) ...
        ||isnan(Data(i).pipe_x(1))||isnan(Data(i).
           pipe_y(1))||isnan(Data(i).pipe_z(1))) %
           remove NaN
    Data(i).t(1)=[];
    Data(i).x(1)=[];
    Data(i).y(1)=[];
    Data(i).z(1)=[];
    Data(i).vx(1)=[];
    Data(i).vy(1)=[];
    Data(i).vz(1)=[];
    Data(i).ax_imu(1)=[];
    Data(i).ay_imu(1)=[];
    Data(i).az_imu(1)=[];
    Data(i).servo_pos(1)=[];
    Data(i).servo_vel(1)=[];
    Data(i).servo_amp(1)=[];
    Data(i).pipe_x(1)=[];
    Data(i).pipe_y(1)=[];
    Data(i).pipe_z(1)=[];
end
while(isnan(Data(i).x(end))||isnan(Data(i).y(end))||
   isnan(Data(i).z(end)) ...
        ||isnan(Data(i).vx(end))||isnan(Data(i).vy(end)
           )||isnan(Data(i).vz(end)) ...
        ||isnan(Data(i).ax_imu(end))||isnan(Data(i).
           ay_imu(end))||isnan(Data(i).az_imu(end))...
```

```
        ||isnan(Data(i).pipe_x(end))||isnan(Data(i).
           pipe_y(end))||isnan(Data(i).pipe_z(end))) %
           remove NaN
    Data(i).t(end)=[];
    Data(i).x(end)=[];
    Data(i).y(end)=[];
    Data(i).z(end)=[];
    Data(i).vx(end)=[];
    Data(i).vy(end)=[];
    Data(i).vz(end)=[];
    Data(i).ax_imu(end)=[];
    Data(i).ay_imu(end)=[];
    Data(i).az_imu(end)=[];
    Data(i).servo_pos(end)=[];
    Data(i).servo_vel(end)=[];
    Data(i).servo_amp(end)=[];
    Data(i).pipe_x(end)=[];
    Data(i).pipe_y(end)=[];
    Data(i).pipe_z(end)=[];
end
%         if strcmp(Data(i).name,'official_test_2021
   -08-27-16-39-40_halfsuccess')
%             figure
%             plot(t,Data(i).FD.z)
%             hold on
%             plot(t_pipe,Data(i).Pipe.z)
%             plot(Data(i).t,Data(i).z)
%             plot(Data(i).t,Data(i).pipe_z)
%         end

%% isolate data to time during perch
index_perch=Data(i).t>=-1.5 & Data(i).t<=1.5;

Data(i).t(~index_perch)=[];
Data(i).x(~index_perch)=[];
Data(i).y(~index_perch)=[];
Data(i).z(~index_perch)=[];
Data(i).vx(~index_perch)=[];
Data(i).vy(~index_perch)=[];
Data(i).vz(~index_perch)=[];
Data(i).ax_imu(~index_perch)=[];
```

```
Data(i).ay_imu(~index_perch)=[];
Data(i).az_imu(~index_perch)=[];
Data(i).servo_pos(~index_perch)=[];
Data(i).servo_vel(~index_perch)=[];
Data(i).servo_amp(~index_perch)=[];
Data(i).pipe_x(~index_perch)=[];
Data(i).pipe_y(~index_perch)=[];
Data(i).pipe_z(~index_perch)=[];

%% isolating data to flight only
%          tmp=find(Data(i).z>Data(i).pipe_z(1),1,'first
   ');
%          Data(i).t(1:tmp)=[];
%          Data(i).x(1:tmp)=[];
%          Data(i).y(1:tmp)=[];
%          Data(i).z(1:tmp)=[];
%          Data(i).vx(1:tmp)=[];
%          Data(i).vy(1:tmp)=[];
%          Data(i).vz(1:tmp)=[];
%          Data(i).ax_imu(1:tmp)=[];
%          Data(i).ay_imu(1:tmp)=[];
%          Data(i).az_imu(1:tmp)=[];
%          Data(i).servo_pos(1:tmp)=[];
%          Data(i).servo_vel(1:tmp)=[];
%          Data(i).servo_amp(1:tmp)=[];
%          Data(i).pipe_x(1:tmp)=[];
%          Data(i).pipe_y(1:tmp)=[];
%          Data(i).pipe_z(1:tmp)=[];
%
%          tmp=find(Data(i).z<0.2,1,'first');
%          Data(i).t(tmp:end)=[];
%          Data(i).x(tmp:end)=[];
%          Data(i).y(tmp:end)=[];
%          Data(i).z(tmp:end)=[];
%          Data(i).vx(tmp:end)=[];
%          Data(i).vy(tmp:end)=[];
%          Data(i).vz(tmp:end)=[];
%          Data(i).ax_imu(tmp:end)=[];
%          Data(i).ay_imu(tmp:end)=[];
%          Data(i).az_imu(tmp:end)=[];
%          Data(i).servo_pos(tmp:end)=[];
```

```
%          Data(i).servo_vel(tmp:end)=[];
%          Data(i).servo_amp(tmp:end)=[];
%          Data(i).pipe_x(tmp:end)=[];
%          Data(i).pipe_y(tmp:end)=[];
%          Data(i).pipe_z(tmp:end)=[];

%% calculate vicon accel using central difference
Data(i).ax=(Data(i).vx(3:end)-Data(i).vx(1:end-2))/(2*
   Data(i).Dt);
Data(i).ay=(Data(i).vy(3:end)-Data(i).vy(1:end-2))/(2*
   Data(i).Dt);
Data(i).az=(Data(i).vz(3:end)-Data(i).vz(1:end-2))/(2*
   Data(i).Dt);
% calculat pipe vz
Data(i).pipe_vz=(Data(i).pipe_z(3:end)-Data(i).pipe_z
   (1:end-2))/(2*Data(i).Dt);

Data(i).t(end)=[];
Data(i).x(end)=[];
Data(i).y(end)=[];
Data(i).z(end)=[];
Data(i).vx(end)=[];
Data(i).vy(end)=[];
Data(i).vz(end)=[];
Data(i).ax_imu(end)=[];
Data(i).ay_imu(end)=[];
Data(i).az_imu(end)=[];
Data(i).servo_pos(end)=[];
Data(i).servo_vel(end)=[];
Data(i).servo_amp(end)=[];
Data(i).pipe_x(end)=[];
Data(i).pipe_y(end)=[];
Data(i).pipe_z(end)=[];

Data(i).t(1)=[];
Data(i).x(1)=[];
Data(i).y(1)=[];
Data(i).z(1)=[];
Data(i).vx(1)=[];
Data(i).vy(1)=[];
Data(i).vz(1)=[];
```

```
Data(i).ax_imu(1)=[];
Data(i).ay_imu(1)=[];
Data(i).az_imu(1)=[];
Data(i).servo_pos(1)=[];
Data(i).servo_vel(1)=[];
Data(i).servo_amp(1)=[];
Data(i).pipe_x(1)=[];
Data(i).pipe_y(1)=[];
Data(i).pipe_z(1)=[];
%% remove post release data
%         j=length(Data(i).servo_pos);
%         %         if i==13
%         %             dummy=input('wait');
%         %         end
%         for k=2:j
%             if  Data(i).servo_amp(k)>4096*2.69 ||
   Data(i).servo_amp(k)<0 ...
%                     || Data(i).servo_vel(k)>70*4096
   || Data(i).servo_vel(k)<-70*4096 ...
%                     || Data(i).servo_pos(k)>256*4096
   || Data(i).servo_pos(k)<-256*4096 % max current =
   4096*2.69 % No load speed 57 rpm at 14.8v 4096 steps
    per revolution
%                 Data(i).servo_amp(k)=0;
%                 Data(i).servo_vel(k)=0;
%                 Data(i).servo_pos(k)=Data(i).
   servo_pos(k-1);
%             end
%         end
%         while Data(i).servo_pos(j)<max(Data(i).
   servo_pos)
%             Data(i).t(end)=[];
%             Data(i).x(end)=[];
%             Data(i).y(end)=[];
%             Data(i).z(end)=[];
%             Data(i).vx(end)=[];
%             Data(i).vy(end)=[];
%             Data(i).vz(end)=[];
%             Data(i).ax_imu(end)=[];
%             Data(i).ay_imu(end)=[];
%             Data(i).az_imu(end)=[];
```

```
%               Data(i).ax(end)=[];
%               Data(i).ay(end)=[];
%               Data(i).az(end)=[];
%               Data(i).servo_pos(end)=[];
%               Data(i).servo_vel(end)=[];
%               Data(i).servo_amp(end)=[];
%               Data(i).pipe_x(end)=[];
%               Data(i).pipe_y(end)=[];
%               Data(i).pipe_z(end)=[];
%               j=j-1;
%           end
%% accel filtering
Data(i).ax_filter(1:3,1)=Data(i).ax(1:3);
Data(i).ay_filter(1:3,1)=Data(i).ay(1:3);
Data(i).az_filter(1:3,1)=Data(i).az(1:3);
Data(i).ax_imufilter(1:3,1)=Data(i).ax_imu(1:3);
Data(i).ay_imufilter(1:3,1)=Data(i).ay_imu(1:3);
Data(i).az_imufilter(1:3,1)=Data(i).az_imu(1:3);
for j=4:length(Data(i).t)
    Data(i).ax_filter(j)=(1+4*Data(i).Dt*wc+2*Data(i).
       Dt^2*wc^2+Data(i).Dt^3*wc^3)^-1 ...
        *(Data(i).Dt^3*wc^3*Data(i).ax(j)+(3+10*Data(i)
           .Dt*wc+2*Data(i).Dt^2*wc^2)*Data(i).
           ax_filter(j-1)-(3+8*Data(i).Dt*wc)*Data(i).
           ax_filter(j-2)+(1+2*Data(i).Dt*wc)*Data(i).
           ax_filter(j-3));
    Data(i).ay_filter(j)=(1+4*Data(i).Dt*wc+2*Data(i).
       Dt^2*wc^2+Data(i).Dt^3*wc^3)^-1 ...
        *(Data(i).Dt^3*wc^3*Data(i).ay(j)+(3+10*Data(i)
           .Dt*wc+2*Data(i).Dt^2*wc^2)*Data(i).
           ay_filter(j-1)-(3+8*Data(i).Dt*wc)*Data(i).
           ay_filter(j-2)+(1+2*Data(i).Dt*wc)*Data(i).
           ay_filter(j-3));
    Data(i).az_filter(j)=(1+4*Data(i).Dt*wc+2*Data(i).
       Dt^2*wc^2+Data(i).Dt^3*wc^3)^-1 ...
        *(Data(i).Dt^3*wc^3*Data(i).az(j)+(3+10*Data(i)
           .Dt*wc+2*Data(i).Dt^2*wc^2)*Data(i).
           az_filter(j-1)-(3+8*Data(i).Dt*wc)*Data(i).
           az_filter(j-2)+(1+2*Data(i).Dt*wc)*Data(i).
           az_filter(j-3));
```

```
    Data(i).ax_imufilter(j)=(1+4*Data(i).Dt*wc+2*Data(i
       ).Dt^2*wc^2+Data(i).Dt^3*wc^3)^-1 ...
        *(Data(i).Dt^3*wc^3*Data(i).ax_imu(j)+(3+10*
           Data(i).Dt*wc+2*Data(i).Dt^2*wc^2)*Data(i).
           ax_imufilter(j-1)-(3+8*Data(i).Dt*wc)*Data(i
           ).ax_imufilter(j-2)+(1+2*Data(i).Dt*wc)*Data
           (i).ax_imufilter(j-3));
    Data(i).ay_imufilter(j)=(1+4*Data(i).Dt*wc+2*Data(i
       ).Dt^2*wc^2+Data(i).Dt^3*wc^3)^-1 ...
        *(Data(i).Dt^3*wc^3*Data(i).ay_imu(j)+(3+10*
           Data(i).Dt*wc+2*Data(i).Dt^2*wc^2)*Data(i).
           ay_imufilter(j-1)-(3+8*Data(i).Dt*wc)*Data(i
           ).ay_imufilter(j-2)+(1+2*Data(i).Dt*wc)*Data
           (i).ay_imufilter(j-3));
    Data(i).az_imufilter(j)=(1+4*Data(i).Dt*wc+2*Data(i
       ).Dt^2*wc^2+Data(i).Dt^3*wc^3)^-1 ...
        *(Data(i).Dt^3*wc^3*Data(i).az_imu(j)+(3+10*
           Data(i).Dt*wc+2*Data(i).Dt^2*wc^2)*Data(i).
           az_imufilter(j-1)-(3+8*Data(i).Dt*wc)*Data(i
           ).az_imufilter(j-2)+(1+2*Data(i).Dt*wc)*Data
           (i).az_imufilter(j-3));
end

%% perch detection
if Data(i).z(Data(i).z==min(Data(i).z))>Data(i).pipe_z(
   Data(i).z==min(Data(i).z))-0.22 & ~contains(Data(i).
   name,'fail')
    Data(i).perch=1;
else
    Data(i).perch=0;
end


%% use max jerk-z as impact
Data(i).jz=zeros(length(Data(i).t),1);
Data(i).jz(2:end)=(Data(i).az_filter(2:end)-Data(i).
   az_filter(1:end-1))/Data(i).Dt;
condition_xy_approx=Data(i).x<(Data(i).pipe_x(1)+0.40)
   & Data(i).x>(Data(i).pipe_x(1)-0.40) & Data(i).y<(
   Data(i).pipe_y(1)+0.20) & Data(i).y>(Data(i).pipe_y
   (1)-0.20);
```

```
        Data(i).index_window_start=find(Data(i).z<(Data(i).
           pipe_z(1)+0.15) & condition_xy_approx,1,'first');
        Data(i).index_window_end=find(Data(i).z>(Data(i).pipe_z
           (1)-0.3) & condition_xy_approx,1,'last');

        %tmp=find(Data(i).jz==max(Data(i).jz));
        Data(i).index_impact=Data(i).jz==max(Data(i).jz(Data(i)
           .index_window_start:Data(i).index_window_end));
        Data(i).index_impact=find(Data(i).index_impact,1,'first
           ')-2; % normally -2

        Data(i).servo_impact=Data(i).servo_pos(Data(i).
           index_impact);
    end
    [~,I]=sort([Data.servo_impact]);
    Data_processed=Data(I);

    processed=1;
    disp('Data processed')
else
    disp('Skip processing')
end
clearvars -except Data_processed Data_raw processed
close all
Data=Data_processed;
outliers=[8 31 32];%[8 14 31 32 33];
Data(14).index_impact=Data(14).index_impact+1;
Data(31).index_impact=Data(31).index_impact+1;
Data(33).index_impact=Data(33).index_impact+1;
figure
for i=1:length(outliers)
    subplot(length(outliers),1,i)
    plot(Data(outliers(i)).t,Data(outliers(i)).z)
    hold on
    plot(Data(outliers(i)).t,Data(outliers(i)).pipe_z)
    plot(Data(outliers(i)).t(Data(outliers(i)).index_impact),
       Data(outliers(i)).pipe_z(Data(outliers(i)).index_impact)
       ,'^')
end

Data(outliers)=[];
```

```
n_data=length(Data);

for i=1:n_data
    drop_y(i)=Data(i).y(Data(i).t==0)-Data(i).pipe_y(1);
    drop_z(i)=Data(i).z(Data(i).t==0)-Data(i).pipe_z(1)+.145/2;
    drop_vz(i)=Data(i).vz(Data(i).t==0);
    impact_y(i)=Data(i).y(Data(i).index_impact)-Data(i).pipe_y
       (1);%-Data(i).pipe_y(Data(i).index_impact);
    impact_z(i)=Data(i).z(Data(i).index_impact)+.145/2-Data(i).
       pipe_z(1);%-Data(i).pipe_z(Data(i).index_impact);
    impact_vz(i)=min(Data(i).vz)-Data(i).pipe_vz(Data(i).
       index_impact);
end

%% x and vz at impact

% figure
% hold on
perch_servo_pos=zeros(size(Data));
perch_success=perch_servo_pos;
for i=1:n_data
    perch_servo_pos(i)=Data(i).servo_pos(Data(i).index_impact);
    if Data(i).perch
        perch_success(i)=true;
        %         a=plot(Data(i).servo_pos(Data(i).index_impact
           ),Data(i).vz(Data(i).index_impact),'o');
        %         a.MarkerEdgeColor=[0.4660 0.6740 0.1880];
        %         a.MarkerFaceColor=[0.4660 0.6740 0.1880];
    end
end
perch_success=logical(perch_success);


%% Plotting accuracy vs success rate
servo_flat=1400; % XH430 0.088 deg per resolution
servo_diff=1650; % difference between close and flat
r_spool=6; % spool radius
l_t=10; % distance between joint and tendon hole

zone_r=100;
gap=10;
```

```
perch_ang_arm=real(180-2*asind((l_t-(perch_servo_pos-servo_diff
   -[Data.servo_init])*0.088/180*pi*r_spool/2)/l_t)); % servo->
   spool angle->tendon retraction->half internal angle->
   external angle
[arm_xs,arm_ys]=pol2cart(perch_ang_arm(perch_success)/180*pi+pi
   ,zone_r+gap);
[arm_xf,arm_yf]=pol2cart(perch_ang_arm(~perch_success)/180*pi+
   pi,zone_r+gap);

d_branch=140;
r_branch=d_branch/2;
x=meshgrid((-95:.05:45)*2); % graph resolution
y=x';
zone=NaN(size(x));
z_zone=[100,75,50,10;5^2,25^2,50^2,70^2];

drone_h=56; % drone body height
drone_w=40; % drone body half width
m0=(-2*drone_w^2*r_branch)/((r_branch^2+drone_w^2)*(2*(-drone_w
   )*r_branch^2/(drone_w^2+r_branch^2)+drone_w)); % arm
   gradient
optimal_arm_ang=atan2d(m0,1);
drone=[-drone_w,-drone_w,drone_w,drone_w,-drone_w*.9,-drone_w
   *.9, drone_w*.9,drone_w*.9,-drone_w*.9,-drone_w*.9,-drone_w;
   drone_h,0,0,drone_h,drone_h,drone_h*.9,drone_h*.9,drone_h
   *.1,drone_h*.1,drone_h,drone_h];
% drone(x>-10&x<10&y>0&y<10)=100;
% drone(x>-8&x<8&y>1.5&y<8.5)=0;
% [drn_idx_x,drn_idx_y]=find(drone~=0);

% % % for radial zone
% % for i=size(z_zone,2):-1:1
% %     z(((x+10).^2+(y).^2)<=z_zone(2,i))=z_zone(1,i);
% % end


% pct_groups=[90,75,60,45,30,15,0,-15,-30,-45,-60,-75,-90]+
   optimal_arm_ang;
pct_groups=[90:-15:-90]+optimal_arm_ang;
success_rate=zeros(3,size(pct_groups,2)-1);
```

```
for i=2:size(pct_groups,2)
    success_rate(1,i-1)=sum(perch_ang_arm>pct_groups(i) &
       perch_ang_arm<=pct_groups(i-1) & perch_success);
    success_rate(2,i-1)=sum(perch_ang_arm>pct_groups(i) &
       perch_ang_arm<=pct_groups(i-1));
    success_rate(3,i-1)=success_rate(1,i-1)/success_rate(2,i-1)
       ;
    if isnan(success_rate(3,i-1))
        success_rate(3,i-1)=0;
    end
end

for i=1:size(success_rate,2)
    zone(atan2d(y,x+drone_w)>=pct_groups(i+1)-180 & atan2d(y,x+
       drone_w)<=pct_groups(i)-180 & ((x+drone_w).^2+(y).^2)<=(
       zone_r/(max(success_rate(2,:))+mod(max(success_rate(2,:)
       ),2))*success_rate(2,i)+gap)^2 & ((x+drone_w).^2+(y).^2)
       >=gap^2)=success_rate(3,i); % high side
end

g1=figure;

contourf(x,y,zone*100)
hold on

% [tx_posx,tx_posy]=pol2cart(([20:-20:-80]+optimal_arm_ang)
   /180*pi+pi,zone_r+20+gap);
% [line_x0,line_y0]=pol2cart(([20:-20:-80]+optimal_arm_ang)
   /180*pi+pi,gap);
% tx=["+20^o","0^o","-20^o","-40^o","-60^o","-80^o"];
[tx_posx,tx_posy]=pol2cart(([30:-30:-90]+optimal_arm_ang)/180*
   pi+pi,zone_r+20+gap);
line_ang=([30:-15:-90]+optimal_arm_ang); % angle of radila
   lines
line_ang(line_ang<0)=0;
[line_x0,line_y0]=pol2cart(line_ang/180*pi+pi,gap); % radial
   lines
[line_x1,line_y1]=pol2cart(line_ang/180*pi+pi,zone_r+15+gap); %
    radial lines
[line_x1(2:2:end),line_y1(2:2:end)]=pol2cart(line_ang(2:2:end)
   /180*pi+pi,zone_r+gap);
```

```
tx=["+30^o","0^o","-30^o","-60^o","-90^o"];
tx(tx_posy>0)=num2str(round(-optimal_arm_ang))+"^o";
line_posx=tx_posx;
line_posy=tx_posy;
line_posx(tx_posy>=0)=tx_posx(tx_posy>=0)*1.2;
line_posy(tx_posy>0)=0;


tx_posy(tx_posy>0)=7;
tx_posx(tx_posy>0)=tx_posx(tx_posy>=0)*1.02;
% b=[0 0 0 0 0 0 0 0 0 0 0]+.2;
% g=[0 0 0 0 0 0 0.2 0.4 0.6 0.8 1];
% r=[1 0.8 0.6 0.4 0.2 0 0 0 0 0 0];

r=[220 220 220 220 220 220 181.2000  142.4000  103.6000
   64.8000   26]/255;
g=[50.0000   76.6000  103.2000  129.8000  156.4 183 173 163 153
    143 133]/255;
b=[32.0000   76.6000  121.2000  165.8000  210.4000  217.8333
   225.2667 232.7000 240.1333 247.5667 255]/255;

map = [
    r(1) g(1)  b(1)
    r(2) g(2)  b(2)
    r(3) g(3)  b(3)
    r(4) g(4)  b(4)
    r(5) g(5)  b(5)
    r(6) g(6)  b(6)
    r(7) g(7)  b(7)
    r(8) g(8)  b(8)
    r(9) g(9)  b(9)
    r(10) g(10) b(10)
    r(11) g(11) b(11)]*.8;


%fill(drone(1,:),drone(2,:),'b') % drone body fill
colormap(g1,map)
%fimplicit(@(x,y) x^2+(y+d_branch/2)^2-(d_branch/2)^2,'k','
   LineWidth',3);
cb = colorbar;
cb.Ruler.TickLabelFormat='%g%%';
```

```
cb.Label.String ='Perch Success Rate';
cb.Label.FontSize = 12;
% cb.Position=[0.75 0.25 0.02 0.5];
cb.Location='northoutside';
l_arm=35*3;
y2=-m0*l_arm/(1+m0^2)^.5;
x2=-l_arm/(1+m0^2)^.5-drone_w;
plot([-drone_w x2],[0 y2],'-','Color',[.2 .2 .2],'LineWidth',2)
    % drone arm


for i=0:2:(max(success_rate(2,:))+mod(max(success_rate(2,:)),2)
   ) % plotting circumferential lines
    th=linspace(0,30+optimal_arm_ang,400*i)/180*pi+pi;
    [x_line,y_line]=pol2cart(th,i/(max(success_rate(2,:))+mod(
       max(success_rate(2,:)),2))*zone_r+gap);
    plot(x_line-drone_w,y_line,'-','Color',[0.75 0.75 0.75],'
       LineWidth',1.25);
end
for i=1:length(line_x0)
    plot([line_x0(i)-drone_w,line_x1(i)-drone_w],[line_y0(i),
       line_y1(i)],'-','Color',[0.5 0.5 0.5],'LineWidth',1.75)
end
[tx1_posx,tx1_posy]=pol2cart(pi,(0:2:(max(success_rate(2,:))+
   mod(max(success_rate(2,:)),2)))/((max(success_rate(2,:))+mod
   (max(success_rate(2,:)),2)))*zone_r+gap);
tx1_numbers=["0";num2str([2:2:(max(success_rate(2,:))+mod(max(
   success_rate(2,:)),2))]')]';
axis equal
axis off

J = imread('drone_fig/sloth_body_v2.png');
Jtp=ones(size(J,1),size(J,2));
Jtp=0.2*(Jtp-all(J==255,3));
J = imrotate(J,180);
Jtp = imrotate(Jtp,180);
image('CData',J,'XData',[-drone_w 2*drone_w*.5-drone_w],'YData'
   ,[0 drone_h],'AlphaData',Jtp)

K = imread('drone_fig/sloth_arm.png');
Ktp=ones(size(K,1),size(K,2));
```

```
Ktp=0.2*(Ktp-all(K==255,3));
K = imrotate(K,-optimal_arm_ang);
Ktp = imrotate(Ktp,-optimal_arm_ang);
K1=flip(K,2);
Ktp1=flip(Ktp,2);
image('CData',K,'XData',[-140*cosd(optimal_arm_ang)-30*sind(
   optimal_arm_ang) 0]*.9-drone_w+6,'YData',[-140*sind(
   optimal_arm_ang) 30*cosd(optimal_arm_ang)]*.9,'AlphaData',
   Ktp)
image('CData',K1,'XData',[0 140*cosd(optimal_arm_ang)+30*sind(
   optimal_arm_ang)]*.9-6,'YData',[-140*sind(optimal_arm_ang)
   30*cosd(optimal_arm_ang)]*.9,'AlphaData',Ktp1)
plot(arm_xs-drone_w,arm_ys,'o','Color',[26 133 255]/255/2,'
   MarkerFaceColor',[26 133 255]/255,'MarkerSize',6)
plot(arm_xf-drone_w,arm_yf,'x','Color',[255 0 0]/255/2,'
   LineWidth',2);
text(tx_posx-drone_w,tx_posy,tx,'HorizontalAlignment','center',
   'FontSize',12) % arm angle
text(tx1_posx-drone_w,tx1_posy+18,tx1_numbers,'
   HorizontalAlignment','center','FontSize',10) % plot "number
   of tests" units
text(tx1_posx(4)-drone_w-2,tx1_posy(4)+35,'Number of Tests','
   HorizontalAlignment','center','FontSize',12)
text(-drone_w,-l_arm-40,"Arm Position (Degree)",'
   HorizontalAlignment','center','FontSize',12)
%imshow(J);
disp("Done 1")


%% error bars for perching success & failure

y_success =(impact_y(perch_success))';
y_fail = (impact_y(~perch_success))';
combined_y=[y_fail;y_success];
u1 = repmat('perch success',length(y_success),1);
u2 = repmat('perch failure',length(y_fail),1);
u = [u2; u1];
g2=figure;
boxplot(combined_y*1000,u,'Notch','on','Orientation','
   horizontal')
```

```
xlabel('Perpendicular distance offset from target (mm)')
% g2.Position = [300 300 180 370];

vz_success =impact_vz(perch_success)';
vz_fail =impact_vz(~perch_success)';
combined_vz=[vz_fail;vz_success];
figure
boxplot(combined_vz,u,'Notch','on','Orientation','vertical');
ylim([-6 -2])
ylabel('impact velocity (m/s)')

figure
ang_success=perch_ang_arm(perch_success)'-optimal_arm_ang;
ang_fail=perch_ang_arm(~perch_success)'-optimal_arm_ang;
ang_success1=ang_success(ang_success<-50);
ang_success2=ang_success(ang_success>=-50);
combined_ang=[ang_success1;ang_success2;ang_success;ang_fail];
u1 = repmat('perch success',length(ang_success),1);
u11= repmat('perch succes1',length(ang_success1),1);
u12= repmat('perch succes2',length(ang_success2),1);
u2 = repmat('perch failure',length(ang_fail),1);
u = [u11;u12;u1;u2];
combined_ang=[ang_success;ang_fail];
u = [u1;u2];
% subplot(2,1,1)
boxplot(combined_ang,u,'Notch','on','Orientation','vertical');
ylim([-inf 0])
ylabel('impact arm angle (deg)')

%% functions
function out = Count(in)
global ig
out=0;
if in(ig) == 0
    out = 0;
else
    ig=ig+1;
    out = out + 1 + Count(in);
end
end
```

Supplementary Table S(2): **Prototype robot component mass breakdown**. The integrated subsystems for perching and flight are highlighted in blue and red, respectively. The overlap of the subsystems is due to the component performing dual roles.

Function group	Components	Mass (g)	
Sensing & Command	Tracking Camera	55.0	
	Computer Voltage Converter	9.0	
	Cables	16.0	
	Flight Computer	54.0	Perching Subsystem, Flight Subsystem
	Flight Controller	9.0	Perching Subsystem, Flight Subsystem
Flight Suite	Battery connector cables	10.0	Perching Subsystem, Flight Subsystem
	2 Motor Cables	24.0	Flight Subsystem
	2 ESC + 2 BLDC Motors + 2 Propellers	67.9	Flight Subsystem
	2 Motor Cables	24.0	Perching Subsystem, Flight Subsystem
	2 ESC + 2 BLDC Motors + 2 Propellers	67.9	Perching Subsystem, Flight Subsystem
Perching Electronics	USB Communication Converter	9.0	Perching Subsystem
	Servomotor	88.0	Perching Subsystem
	Peripheral Electronics	12.0	Perching Subsystem
	Mounts	2.5	Perching Subsystem
Structure	Morphing Arms	48.0	Perching Subsystem, Flight Subsystem
	Carbon Fiber Body	58.8	Perching Subsystem, Flight Subsystem
	Static Arms	26.0	Flight Subsystem
	Nuts/Bolts/Standoffs	40.0	Flight Subsystem
	TPU Computer Mount + Bumper	72.0	
	TPU Camera Mount	11.0	
	Vicon Bubble + Mounts	12.0	
Battery	4 Cell LiPo	148.0	
	Battery alarm	8.0	
Subtotal	Perching Subsystem		383.2
	Flight Subsystem		429.5
Total	w/o batt/batt alarm/camera	650.0	
	w/ batt	872.0	
